# Methodologies and Wearable Devices to Monitor Biophysical Parameters Related to Sleep Dysfunctions: An Overview

**DOI:** 10.3390/mi13081335

**Published:** 2022-08-17

**Authors:** Roberto De Fazio, Veronica Mattei, Bassam Al-Naami, Massimo De Vittorio, Paolo Visconti

**Affiliations:** 1 Department of Innovation Engineering, University of Salento, 73100 Lecce, Italy; 2 Department of Biomedical Engineering, Faculty of Engineering, The Hashemite University, Zarqa 13133, Jordan; 3 Center for Biomolecular Nanotechnologies, Italian Technology Institute IIT, 73010 Arnesano, Italy

**Keywords:** sleep dysfunction, sensors, wearable devices, EEG, ECG, EOG, polysomnography, sleep staging

## Abstract

Sleep is crucial for human health from metabolic, mental, emotional, and social points of view; obtaining good sleep in terms of quality and duration is fundamental for maintaining a good life quality. Over the years, several systems have been proposed in the scientific literature and on the market to derive metrics used to quantify sleep quality as well as detect sleep disturbances and disorders. In this field, wearable systems have an important role in the discreet, accurate, and long-term detection of biophysical markers useful to determine sleep quality. This paper presents the current state-of-the-art wearable systems and software tools for sleep staging and detecting sleep disorders and dysfunctions. At first, the paper discusses sleep’s functions and the importance of monitoring sleep to detect eventual sleep disturbance and disorders. Afterward, an overview of prototype and commercial headband-like wearable devices to monitor sleep is presented, both reported in the scientific literature and on the market, allowing unobtrusive and accurate detection of sleep quality markers. Furthermore, a survey of scientific works related the effect of the COVID-19 pandemic on sleep functions, attributable to both infection and lifestyle changes. In addition, a survey of algorithms for sleep staging and detecting sleep disorders is introduced based on an analysis of single or multiple biosignals (EEG—electroencephalography, ECG—electrocardiography, EMG—electromyography, EOG—electrooculography, etc.). Lastly, comparative analyses and insights are provided to determine the future trends related to sleep monitoring systems.

## 1. Introduction

Sleep is a fundamental biological process for human life, as it plays a fundamental role in maintaining physical, psychological, emotional, and social health thanks to its restorative, regenerative, conservative, consolidating, and protective functions [1,2,3]. In fact, sleep’s poor quality or its insufficient duration can have both short- and long-term repercussions, such as a decrease in life’s quality, difficulty in concentrating, learning problems, bad mood, excessive daytime sleepiness, accidents of various types, weakening of the immune system, propensity to contract infections and diseases, etc. [4,5,6]. Numerous negative health impacts, such as an elevated risk of hypertension, diabetes, obesity, depression, heart attack, and stroke, have been associated with the cumulative long-term effects of sleep disturbances and sleep deprivation [7]. The most common sleep disorders, according to their prevalence, are the following: insomnia, sleep apnea, restless legs syndrome (RLS), hypersomnia, parasomnia, circadian rhythm disorders, obstructive sleep apnea, nocturnal, epilepsy, etc. [8,9,10,11]. Therefore, monitoring sleep and related disorders is crucial in preventing diseases [12,13,14].

Moreover, sleep health is a complex sleep-wakefulness pattern adaptable to individual, social, and environmental needs and supports physical and mental health [15]. Subjective satisfaction, optimal timing, adequate duration, high efficiency, and maintained attentiveness throughout waking hours are characteristics of healthy sleep. Thus, quantifying sleep health requires a set of measurable quantities related to physical, mental, and neurobehavioral well-being. These lasts can be classified into five dimensions: sleep duration, continuity, timing, alertness, sleepness, and satisfaction/quality. Some of them are based on subjective evaluation (e.g., satisfaction/quality); thus, sleep health can be quantified through subjective reports.

Wearable devices offer a valuable contribution in this area, as they allow collection, analysis, and transmission during sleep of vital and functional parameters, including body temperature, heart rate, respiratory rate, blood oxygen saturation, etc. [16,17,18,19,20,21]. Furthermore, these solutions are discreet and not bulky (contrary to the polysomnography exam, which remains the gold standard for recording electroencephalographic, respiratory, cardiac, and eye movements activities) and allow sleep monitoring even from home. Moreover, the recent spread of COVID-19 had a negative impact on sleep due to increased anxiety, insecurity, stress, fear, loneliness, depression, etc. These sleep alterations affect a wide portion of the global population, not only subjects who tested positive for this infection [22,23,24].

Therefore, this paper aims to analyze the state-of-the-art headband-like wearable devices applied to the head to identify the best solution that balances accuracy in parameter detection and comfort [25,26]. We have focused on the devices applied to the head as they can acquire a set of parameters similar to that of polysomnography, mainly the EEG, carrying out multiple information about the user’s brain activity during sleep. In particular, both research works and commercial devices were analyzed and discussed to evaluate the state of development of the scientific community in this area. Both hardware and firmware sections of discussed wearable devices have been thorough, highlighting the strengths and weaknesses of discussed solutions. Afterward, a survey of conventional and machine learning algorithms and software tools for sleep staging and detecting sleep disorders based on the processing of single or multiple biosignals (e.g., EEG—electroencephalography, ECG—electrocardiography, EMG—electromyography, and/or EOG—electrooculography). Comparative analyses are provided in every section, providing insights for determining the best solutions for sleep staging and disorder detection. Finally, an overview of research works dealing with the consequences on the sleep of the COVID-19 pandemic is presented, ascribable to both effects of infections on the human body and change of habits.

The main contributions of the proposed paper are:

- An in-depth discussion about sleep’s role in psychological, physiological, and behavioral mechanisms and the importance of sleep monitoring to detect and track symptoms related to sleep disturbances or disorders;
- A comprehensive overview of prototype and commercial wearable devices for sleep monitoring, reported in the scientific literature and on the market, enabling discreet and accurate monitoring of users’ vital signs during sleep;
- An overview of classical and representative-learning algorithms for sleep staging and sleep disorder detection by analyzing data related to single or multiple vital signs;
- A survey of the scientific works analyzing the effect of the COVID-19 pandemic on sleep functions, ascribable to both infection and changes in lifestyle.

The review paper presented here differs from similar ones as it treats both commercial and prototype devices discussed in the literature, giving the reader a more comprehensive understanding of the subject. In addition, the presented article separately discusses wearable devices and algorithms for sleep classification and sleep dysfunction detection, allowing for a more in-depth analysis of the individual issues. In contrast, other review works provide a unified discussion of devices and algorithms, not providing a detailed discussion of the architecture, mode of operation, and performance of the presented devices. Finally, our work discusses the consequences of the COVID-19 pandemic on sleep due to infection and lifestyle changes; we believe this to be an added value since, to our knowledge, no review paper discusses this topic in the literature.

The paper is arranged as follows: Section 2 deals with the function of sleep in psychophysiological and behavioral mechanisms and the importance of monitoring the sleep stage and detecting eventual sleep disorders/disturbances. Then, the important role of wearable devices is discussed for continuous and non-invasive sleep monitoring that does not require the presence of qualified technicians. Thus, Section 3 proposes an overview of wearable devices reported in scientific literature. Afterward, Section 4 introduces a survey of wearable devices available on the market. The goal is to determine the best solutions, taking into account the type and number of parameters detected, as well as the possible availability of a wireless module for real-time data transfer and invasiveness (for commercial devices, even the cost). Section 5 focuses on conventional and deep learning algorithms that can be used to analyze data collected through various sensors and extract features (essential for the sleep stages classification and sleep disorders detection). Finally, the effects of the COVID-19 pandemic on sleep are discussed, reporting a clinical case concerning a male subject with mild infection.

## 2. Importance of Sleep Monitoring

This section outlines an overview of sleep mechanisms and related disorders, stressing the importance of sleep monitoring to prevent such diseases [27,28,29]. Sleep is characterized scientifically by the individual behavior while asleep and the accompanying physiological changes occurring in the waking brain’s electrical cycles during sleep. A reduction or lack of mobility, slow eye movements, a distinctive sleeping body posture, lower reactivity to external stimuli, longer reaction time, heightened arousal threshold, diminished cognitive function, and unconscious but reversible state are the behavioral phenomena featuring sleep. Moreover, physiological changes may be measured using specific clinical tests involving the acquisition of several biosignals, such as EEG, EOG, EMG, or ECG.

**Figure 1:**
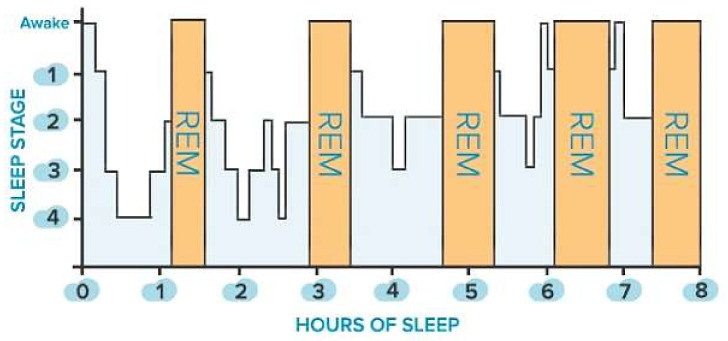
Sleep stages of a healthy adult: non-rapid eye movement (NREM, blue bars) and rapid eye movement (REM, orange bars) stages.

Sleep is classified into two main states: non-rapid eye movement (NREM) sleep, which is distinguished into three stages (i.e., N1, N2, N3), and rapid eye movement (REM) sleep. The first is defined by a gradual decrease in reactivity to stimulus, slow rolling eye movements, slightly reduced mobility, reversible unconscious state, and synchronized EEG characterized by slow-wave activity associated with spindles and K-complexes, as well as diminished muscular tone. The latter is characterized by fast eye movements, reduced reactivity to stimulus, muscular atonia, fast rhythms, and myoclonic jerks. In addition, other symptoms are desynchronized EEG featured by theta waves, periodic swings in blood pressure and heart rhythm, irregular respiration, phasic tongue movements, and may include a few episodes of apnea or hypopnea [27]. The two states alternate in a cyclic way (from 4 to 6 cycles during sleep, each lasting from 90 to 110 min, Figure 1). From 20% to 25% of total sleep duration in adults is spent in REM sleep, while the remaining 75–80% is spent in NREM sleep. In a healthy adult, there is a gradual progression from wakefulness to sleep onset, NREM sleep, and REM sleep [27]. Nevertheless, this trend is heavily affected by the nervous system’s maturation, changing from childhood to adulthood, as discussed in [27]. The hypothalamus’s suprachiasmatic nuclei master clock regulates the sleep-and-wake circadian rhythm. In addition, the neuroanatomical substrates of NREM sleep are primarily found in the ventrolateral preoptic nucleus of the hypothalamus, while those of REM sleep are found in the pons.

Functional anomalies in the autonomic and somatic nervous systems can induce significant repercussions on sleep, often resulting in sleep disorders. These lasts can be classified into two distinct categories: dyssomnias and parasomnia. The first category comprises the disorders that cause trouble falling or staying asleep, such as insomnia, obstructive sleep apnea, RLS, periodic limb movement, and circadian rhythm disorders. In contrast, the second category includes disorders involving irregular actions executed during sleep, such as arousal disorders, parasomnias associated with REM sleep, and enuresis [30]. It is known that sleep is essential since its functions are assumed to be restorative, conservative, adaptive, thermoregulatory, and memory consolidative. Therefore, sleep deprivation has short and long-term negative repercussions, whether caused by lifestyle or sleep disorders. Short-term consequences include diminished attention and focus, decreased quality of life, higher absenteeism with lower productivity, and workplace, home, and road accidents. Long-term consequences include increased morbidity and mortality from increasing automotive accidents, coronary artery disease, heart failure, high blood pressure, obesity, type 2 diabetes mellitus, stroke, cognitive impairment, and depression. Nevertheless, long-term and short-term repercussions are still a research topic.

Good sleep quality and duration are very important for astronauts, who, instead, often suffer from sleep disorders due to microgravity, isolation, monotonous repetition, workload, low temperatures, noise, interruption of sleep for operational necessities, irregular working schedule, reduced homeostatic regulation, etc. [31]. Among the problems deriving from a poor duration and quality of sleep, it is possible to identify reduced cognitive performance, reduction in concentration and motivation, stress, depression, anxiety, cardiovascular deconditioning, muscle atrophy, and space adaptation syndrome (SAS) as well as a decreased immune response [32,33]. For this reason, NASA (National Aeronautics and Space Administration) has added sleep deprivation among the risk factors that can compromise the cognitive and operational performance and health of astronauts and the safety of missions in orbit. Researchers and scientists are still studying the countermeasures that can be used to avoid the mentioned problems. In [31], some countermeasures for sleep disorders have been proposed, such as creating a good environment in the cabin, adjusting work-rest schedules appropriately, training crew members, intervening pharmacologically, carrying out light treatment, providing adequate psychological support, practicing Tai Chi and using TCM (traditional Chinese medicine).

PSG, which stands for polysomnography, is the gold standard for sleep monitoring, recording several physiologic parameters related to sleep [31]. PSG involves the acquisition of several vital signs, such as EEG, EOG, ECG, EMG, and anterior tibialis EMG, respiratory effort, airflow, and pulse oximetry (i.e., the amount of oxygen in the blood). Electrodermal activity (EDA) is an excellent indicator of the sympathetic nerve’s variations, affected by the sweat glands and blood vessels in the skin. EDA can be a useful tool for describing the various sleep phases compared to other physiological measurements. In detail, the REM phase is featured by high sympathetic nerve activity combined with low muscle tone [34]; moreover, low sympathetic activity is associated with the NREM phase [35,36]. EDA can also be efficiently and reliably acquired using wrist wearable devices during the sleep and wake stages [37]. The collected data can be provided as input for a machine learning algorithm to identify the sleep stage.

## 3. Survey of Wearable Devices for Sleep Monitoring Presented in the Literature

PSG is the reference tool for diagnosing sleep disorders and objectively analyzing the sleep macro and micro-architecture; however, this clinical test requires very bulky clinical instruments. On the other hand, it necessitates a significant number of channels and a large number of electrodes connected to the skin, often causing skin irritation [38]. Furthermore, the electrodes dry out over time, causing a reduction in signal quality. Moreover, electrodes in a PSG monitoring are usually set up by a qualified technician, limiting the possibility of taking these recordings in a person’s home.

However, scientific groups and companies developed a new category of wearable devices and accessories, such as eyewear, masks, devices positioned on the nose, etc., to track sleep and determine its quality. In [39], C. Beach et al. proposed a new electrooculogram device that may be worn to track eye movements. The authors integrated into a traditional eye mask, kept in place merely by the conventional elastic strap, a sensing electrode made by conductive graphene-based textiles from nylon, simply sewing nylon fabric into the unmodified mask using non-conductive polyester thread. Because the device is similar to a classic sleep mask, the device is comfortable and discreet [40]. The mask uses only two EOG channels to classify sleep: the first includes two horizontal electrodes on either side of the eyes, and the second consists of a reference electrode on the forehead. As a final remark, the mask has been tested on four people, demonstrating that the textile graphene-based electrodes sewed inside a traditional sleep mask can be used to track eye movement and successfully detect eye blinks with a signal-to-noise ratio (SNR) above 16.5 dB.

In [41], the authors presented the development of a soft and textile-based sensor platform, called PhyMask, which can reliably measure all of the signals needed for precise sleep stage tracking, as well as extract spindles and K-complexes sleep markers without sacrificing comfort. PhyMask can accurately detect:

- Brain activity using EEG with a high SNR, measuring also advanced sleep markers, such as sleep spindles and K-complexes;
- Eye movement using EOG;
- Physiological factors, with high accuracy for measuring heart rate (median error of 1.7 beats/min) and respiration (median error of 1 BrPM);
- Sleep stages, almost as well as polysomnography;
- Gross body movement and sleep posture.

In detail, PhyMask electrodes should be placed closer to the brain area to obtain significant EEG signals. As a result, an electrode is placed on each side of the head, and one in the center, which serves both as a reference and common ground. In addition, they use fabric-based pressure sensors to monitor the pressure produced by blood pulsing through the facial artery and the small head vibrations caused by blood pumping and respiration over a greater surface area. EOG and EEG signals are provided by biopotential electrodes and cardiorespiratory signals by pressure sensors. Experimental tests were conducted comparing the sleep staging provided by PhyMask with those provided by commercial devices (Fitbit and Oura ring), demonstrating an F1 score of 0.91 using polysomnography as ground truth. The test results demonstrated that the proposed device could accurately detect EEG spindles and K-complexes events, obtaining Cohen’s kappa of sensitivity, specificity, and accuracy higher than 0.8 [41]. In [42], I. Hussain et al. presented a low-cost, portable, and wearable EEG system as a possible prediction tool for stroke onset and post-stroke clinical care. The HealthSOS system consists of an eye mask embedded portable EEG device, an application programming interface (API, based on Java), the networking, signal processing, and machine learning modules, knowledgebase, and a medical health advisor service. The eye mask integrated electrode system and an EEG control module to collect EEG data in the resting state. Therefore, the eye mask includes two dry gold-plated convex EEG electrodes positioned at the frontal cortex Fp1 and Fp2, the best location for recording brainwaves. The open-source OpenBCI Cyton Board (manufactured by OpenBCI Inc., New York, NY, USA) captures EEG signals in portable EEG devices, sending them to a mini-computer through Bluetooth Low Energy (BLE) module. The most important markers for early stroke prediction are revised brain symmetry index (rsBSI), delta-alpha ratio, and delta-theta ratio, used as features for a machine learning algorithm to infer the stroke onset. In particular, the authors employed the support vector machine (SVM) model for detecting the stroke events starting from a set of 48 EEG features; the used model reaches a 92% overall accuracy in stroke prediction.

Many disorders (e.g., stroke, epilepsy, and Parkinson’s disease) can be associated with major abnormal events during sleep. Thus, in [43], the authors have proposed a sleep mask with multiple integrated physiological sensors for collecting and aggregating physiological inputs and a mobile app for data storage, signal processing, and analytics. The mask includes a high-sensitivity microphone as the acoustic sensor to monitor the breathing sound, a triaxial accelerometer to detect head movements and position during sleep, and an eye motion sensor to detect any eye movements. Furthermore, in [44], the authors presented Chesma, a comfortable and lightweight mask that detects physiological signals, such as eye and cardiac parameters, from the face, ensuring good accuracy. It has integrated an ion-conductive fabric pressure sensor that detects pulse waveforms and five hydrogel electrodes (tAgTrode) that wirelessly track eye movements. Four tAgTrodes are located around both eye sockets and the last on the center of the forehead. After acquiring the signals, they are processed and, using a BLE transmission module, sent to a computer every 10 ms. The experiment showed a linear relationship between the eye’s movement and the voltage value measured by the EOG. The authors suggest that the amplitude and frequency of eye movements change as the user transits from awake to deep sleep. In addition, the mask could find application in studies of biomarkers for sleep disorders related to neurodegenerative diseases, such as REM sleep behavior disorder, epilepsy event detection and stroke.

In [45], the authors proposed a new device, ARAM (apnea and rest analysis mask), for monitoring obstructive sleep apnea syndrome (OSA syndrome), which can be used at home without the help of a qualified technician. The ARAM tracks the breathing process through a simple nasal cannula for measuring airflow to detect OSA events and behaviors during sleep (movements and noise) through a microphone and an accelerometer.

Another wearable system, called Morfea, for comfortably detecting sleep apnea and hypopnea events at home is presented in [46]. It is a tiny nasal device that does not use electrodes or cables, so it is comfortable, unobtrusive, lightweight, and does not disturb sleep. It comprises an integrated photoplethysmography (PPG) and a triaxial accelerometer, a microcontroller, and a BLE transmission module to acquire data and then transmit them to a PC for displaying the acquired data in real time. Two methodologies have been implemented, the first based on the power spectral density (PSD) and the second on the pulse wave amplitude (PWA) of the signal, both of which generates a flag (=0 if there are no apnea events, =1 if any). The final output will result from an OR operation between the two flags. Finally, more than 500 apnea and hypopnea episodes were detected, classifying them indiscriminately under the common name apnea, providing an AHI (Apnea-Hypopnea Index) parameter for quantifying the apnea severity, defined as the number of apnea events in the monitoring time. The results demonstrated that Morfea reaches an 89% sensitivity and 93% accuracy in detecting the apnea events.

Table 1 summarizes the scientific works previously discussed, classifying them from the point of view of the number and typology of detected parameters, availability of wireless communication modules, and invasiveness evaluated according to the authors’opinion.

As a result, PhyMask is the device that offers the best balance of comfort and accuracy while tracking sleep parameters. It detects the highest number of parameters compared to the other analyzed devices and provides the opportunity to transfer data through a wireless module. However, the Chesma, with its five hydrogel electrodes, enables an excellent SNR (18.31 dB against −2.62 dB obtained with dry electrodes), allowing accurate tracking of the eye movements and heart parameters also in conditions in which motion artifacts are present. In addition, the two acquired information can be usefully processed for both sleep staging and sleep disorder detection [47,48].

## 4. Overview of Commercial Wearable Devices for Detecting Sleep Disorders

After introducing several wearable devices for sleep monitoring proposed in the scientific literature, this section will illustrate an overview of commercial devices on the market. The most popular devices put comfort ahead of precision. They are not well-adapted to monitor sleep disorders because they solely use heart rate, breathing, and body movement measures to determine sleep quality. In fact, these devices are aimed at healthy users, but they cannot be considered sufficiently reliable for diagnostic sleep disorders.

**Figure 2:**
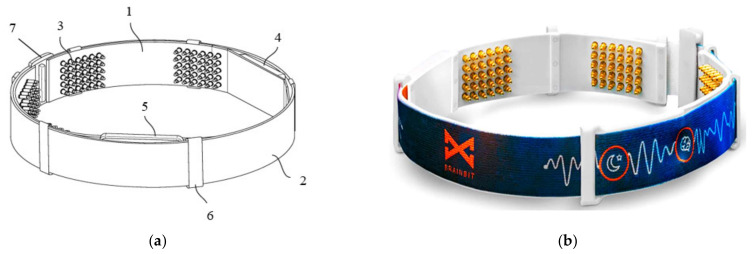
Schematic (**a**) and real (**b**) views of BrainBit (1: ribbon, 2: elastic band, 3: electrodes, 4: electronic module, 5: removable battery, 6: eyelet, 7: clasp) [49].

In [49], Goldstein et al., presented a device called BrainBit, which can be placed on a user’s forehead to track sleep. The device has four EEG channels, with mechanisms for removing EMG and EOG signal components to monitor the electrical brain activity and bioelectricity of the forehead, face, head, and eye muscles [50]. The headband is composed of flexible and non-stretchable ribbon on which the golden dry electrodes, the battery, an integrated electronic board, and an elastic fabric band are placed, as shown in Figure 2.

The four electrodes are placed on the temporal (positions T3 and T4) and occipital (positions O1 and O2) lobe regions; in contrast, the reference sensor is positioned on the forehead. Using the BLE technology, BrainBit transmits the collected biological signals to a PC or a mobile app, where software filters the data and obtains the signals’ spectrum. The great disadvantage of dry electrodes is the rapid degradation of their contact impedance, thus worsening the signal quality [51].

Another commercial device to monitor sleep is Philips’ SmartSleep [52]; it is a lightweight and comfortable wearable sleep headband but not suitable for people with sleep disorders. SmartSleep is also used to track astronauts’ sleep and improve its quality. The device is an EEG-based smart mask to detect deep sleep conditions based on the EEG signal in real-time. When the integrated sensors detect the user in the deep sleep phase, the device emits audio tones to increase the slow waves that characterize this sleep stage. During night use, the device has Bluetooth and Wi-Fi modules turned off. Only when the consumer no longer wears it, this last sends the data to the smartphone app, deriving and visualizing the sleep metrics, such as the amount of time in deep sleep, the time spent falling asleep, the number of times where sleep is interrupted, time spent awake, etc. This operation modality is the major limitation of such a device, especially if real-time sleep quality and staging supervision is required. The device consists of a fabric band, a wet sensor placed behind the right ear (up to three times), and two embedded speakers.

Muse S (Gen 2), in [53], is a headband that allows collecting EEG data and heart parameters (HR and HRV) during sleep, using silver electrodes and a PPG (Photoplethysmographic) sensor, as well as keeping track of the time spent in each stage of sleep, detecting the body position and respiratory activity through inertial sensors. The device is also equipped with “digital sleeping pills” (DSP), which emit immersive sound signals designed to help users fall asleep or fall back asleep if sleep has been interrupted. The device can be used for up to 10 consecutive hours. The fabric headband is lightweight (i.e., only 41 g) and fits different head sizes (from 48 to 63 cm in circumference). The device transmits data via Bluetooth 4.2 to the Muse app. A problem with this device lies in the electrodes’ flat conformation, which could lead to incorrect readings when the area of the body has hair. Tests were conducted to verify the device’s performance, comparing the Muse S to laboratory monitoring devices. Tests have shown that the commercial device can provide detailed and accurate information. A recent 2021 study led by Western University, Cambridge Brain Science, Hatch, and Interaxon that used Muse S EEG-sleep support technology showed a 20% improvement in the Pittsburgh Sleep Quality Index relative to controls wanting to improve their sleep [54].

In [55], Dreem 2 is presented; it allows measuring brain activity, heart rate, respiratory rate, and body movement. The device is a headband that allows the screening, diagnosis, and treatment of sleep disorders directly at the user’s home. The headband integrates six custom-designed EEG electrodes (four frontal and two rear) to monitor brain activity, a PPG sensor to monitor the HR discreetly, and an accelerometer to detect head movements and breathing patterns. In detail, the Dreem 2 can detect the breathing frequency by analyzing the real-time audio recorded during the night by an integrated microphone. However, this detection method is sensitive to the background and glitches, which proper filtering techniques can overcome [56].

**Figure 3:**
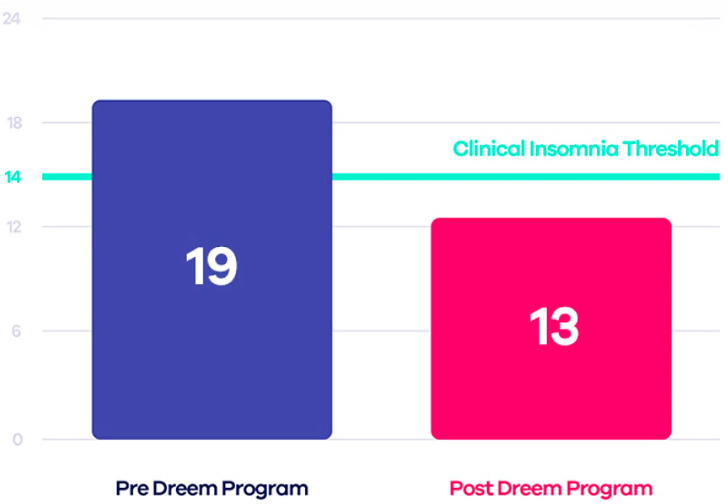
Comparison between ISI scores before and after using Dreem 2 program [55].

Some tests have compared this device to PSG, demonstrating that Dreem 2 is reliable and, therefore, a valid alternative to laboratory tests. The Insomnia Severity Index (ISI) was estimated both before and after using the device to evaluate the effectiveness of Dreem 2 in curing insomnia. The authors applied the CBT-I (Cognitive Behavioral Therapy for Insomnia) exercises and guidelines to design an effective in-app digital program that, when used with the Dreem headband, can encourage patients to take the necessary steps toward healthier sleep. The results demonstrated that 71% of users no longer have insomnia, 91% fall asleep easier taking 21 min less to fall asleep, and 78% wake up fewer times during sleep (Figure 3) [57].

iBand+, presented in [58], is an AI-based (artificial intelligence) headband for detecting EEG signals and head movements. It consists of two stereo speakers placed under the pillow and RGB LEDs (light-emitting diodes) on the headband. The device helps develop lucid dreaming discipline, improve sleep quality, and wake sleeping users gradually. In a lucid dream, the dreamer is aware that he is dreaming and can take control of it without waking up. With the brainwave entrainment technique, which is a method to sync brainwaves with a particular steady rhythmic stimulus, iBand+ helps the user’s brain associate some pulsing sounds and flickering light stimulus with dreams. Lucid dreaming discipline can help users decrease nightmares and stress, improving concentration and creativity. The device also tracks sleep stages, and once the user has fallen asleep, it turns off audiovisual signals and emits white noise, improving sleep quality. In the morning, through a smart alarm, the headband wakes the user up by emitting sounds during the light sleep stage.

**Figure 4:**
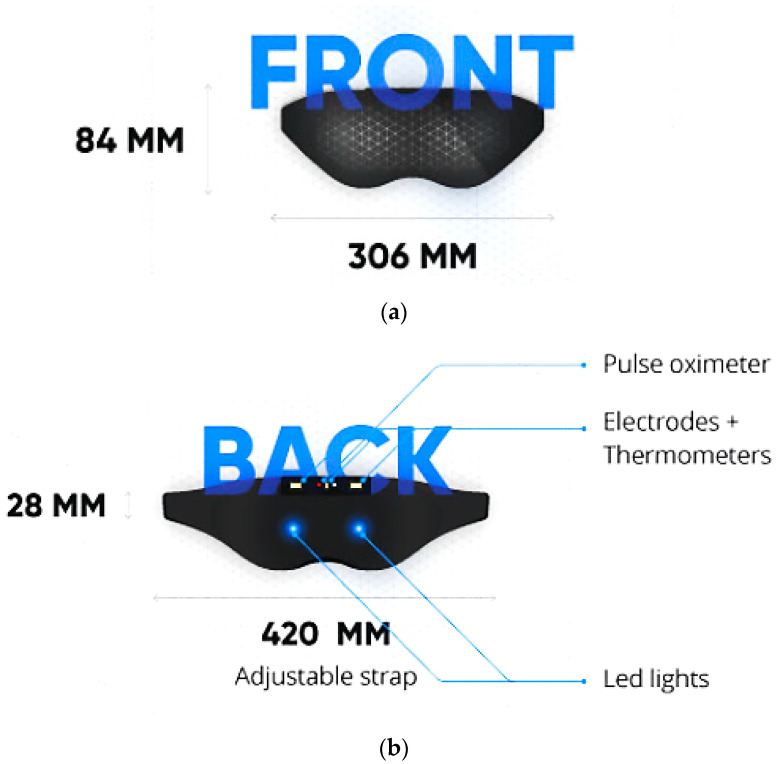
Front (**a**) and rear (**b**) views of Neuroon Open mask [59].

Neuroon Open, in [59], is a wearable mask developed by a Poland startup that detects EEG and EOG signals, blood oxygenation (through PPG sensor), and body temperature; it helps users wake up full of energy and facilitates lucid dreams. Indeed, these data are processed by a suitable sleep staging algorithm run on a custom mobile application; it also calculates the sleep efficiency and provides useful suggestions to enhance sleep habits to obtain a better night’s sleep. The device consists of an adjustable strap with a pulse oximeter, electrodes, EEG and EOG sensors, thermometers, LEDs, and a triaxial accelerometer (Figure 4).

Through BLE 4.0 (designed and marketed by Bluetooth Special Interest Group—BSIG, Kirkland, WA, USA), collected data are transmitted to a mobile device, where Neuroon app allows users to check their sleep analysis (also in real-time) and read personalized tips on improving sleep quality. In addition, through Neuroon app, the users can measure their subjective evaluation of sleep quality every morning. They are given an A/B test, and the combination of self-evaluation and physiological data provides information about the best sleep pattern that suits them. Moreover, this device can help users induce lucid dreaming through light, vibration, and acoustic stimuli and wake up naturally by simulating the light of gradually rising dawn. If the user does not wake up, the device adds vibrations and an audio signal from the mobile phone.

**Figure 5:**
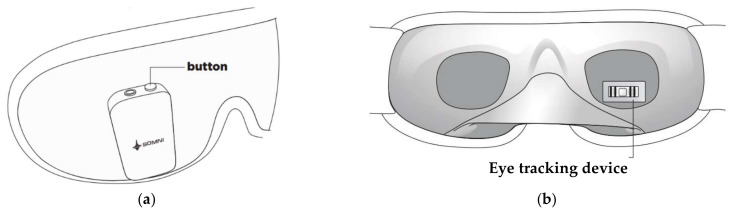
External (**a**) and internal (**b**) views of the Somni mask [60].

Also, the Somni mask, developed by Somni Ltd. (Delft, The Netherlands) startup, monitors sleep by tracking the eye and head movements [60]. Furthermore, the device should create lucid dreams without disturbing sleep by emitting auditory and visual signals. It is composed of a fabric mask similar to conventional ones and, on it, there is an electronic section with a button to turn the device on/off and test the light signals, audio input, and a micro-USB input (Figure 5a). It is not equipped with speakers, so users must connect the headphones to hear the soothing melodies. In addition, the device does not have a wireless module to transfer data, but it can be connected to a PC or a USB OTG (On-The-Go) interface. Inside the mask, an eye-tracking device and accelerometer detect head movements (Figure 5b). However, the headband design may not be comfortable for the user since it has an electronic section positioned in the front part of the mask, causing a feeling of weight on the face.

BrainLink Pro headset, manufactured by Shenzhen Macrotellect Ltd. (Shenzhen, China), monitors brain activity, heart rate, body temperature, and head movements [61]. It consists of three dry metal electrodes, a filter, an amplifier, an A/D converter, a lithium battery lasting 3–4 h, and an adjustable headband that also fits children’s heads. The headband is connected to a smartphone or computer via the BLE 4.0 protocol. The device includes a multi-user EEG acquisition/processing/analysis section called Mindmesh, comprising a Bluetooth gateway, MindMesh EEG sensors, and a front-end acquisition system, allowing the concurrent acquisition of brainwaves of up 50 users. As a result, the device acquires signals with a sampling frequency of 512 Hz, converting them with a 12-bit ADC, ensuring 0.25 µV precision in the signal acquisition and less than 2 ms dynamic response. The device is not oriented to sleep analysis but allows non-invasive detection of EEG signals, which can be used to monitor sleep. Among the device limitations, similarly to other devices previously discussed, the use of dry flat electrodes leads to problems related to contact impedance and applications in hairy body areas. Furthermore, this headband integrates a reference electrode applied to the ear by a clip, which in the long term could create discomfort and irritation to the user.

Sleep Shepherd is a breathable, lightweight, and comfortable headband manufactured by Sleep Shepherd LLC to track head movements, monitor brain activity during sleep, and improve quality [62]. It guides the user toward deep sleep through some binaural beats, played into each ear with two different frequencies. Changing binaural beats’ frequency according to the actual cerebral activity, the device can also wake the user up gradually and during the lightest sleep stage. It comprises EEG sensors and uses NeuroSky’s brain signal filtering technology to remove noises; it also includes a gyroscope and movement sensor for monitoring the user’s movements. The device can be connected wirelessly to a smartphone to display the data collected during the night, such as the duration of sleep, the duration of the deep one, the head movements, the number of times the sleep was interrupted, the time at which the user fell asleep and woke up, etc. However, the device does not stage sleep according to the five standard phases previously described (wake, N1, N2, N3, and REM) but only discerns between awake, light, and deep sleep. Tests were conducted on four healthy awake participants to quantify the accuracy of the NeuroSky sensors and SVM classifier. The participants were divided according to information, mild concentration, and background noise [63]. As a result, classifying the information from noise or mild concentration, the developed system demonstrated high accuracy, with an average of about 86.76.

**Figure 6:**
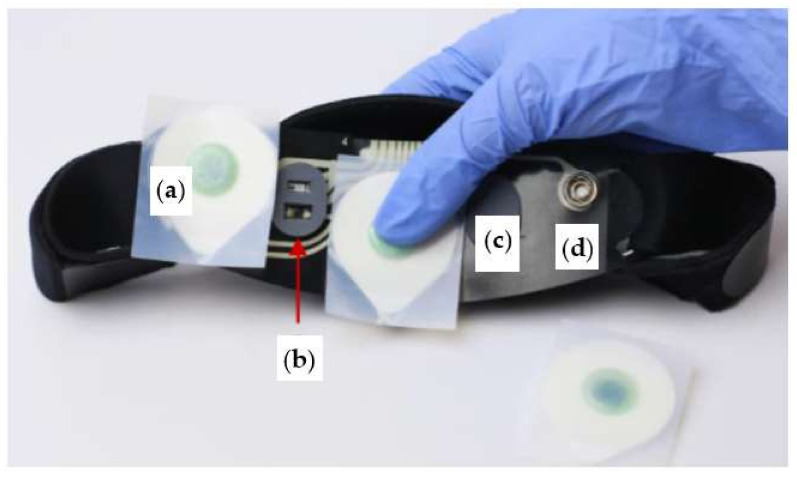
Internal view of Sleep Profiler headband, consisting of EEG sensor (**a**), optical sensor (**b**), strip pad (**c**), and sensor snap (**d**) [64].

In [64], Advanced Brain Monitoring Inc. presents the Sleep Profiler headset that monitors the EEG, EOG, and EMG signals and, optionally, ECG, PPG, head position, or quantitative snoring parameters, using a microphone and an accelerometer (Figure 6). In addition, the model SP29 includes a nasal transducer, a cannula, a pulse rate sensor, and an oximeter. The device is very precise, and it can detect sleep spindles (recording their duration and PSD), distinguish the stages of sleep, identify irregular sleep patterns, and monitor insomnia, hypersomnia, and other sleep disorders. Researchers also use it to evaluate dementia, consequences on sleep due to drug use, effects on memory due to poor sleep quality, etc. [64]; however, it cannot replace clinical ECG or EMG. The Sleep Profiler is very lightweight (71 g) and has up to 8 channels, an internal memory of 8 Gb that can record signals over 10–16 h, and a battery lasting about 30 h. The device uses a speaker to send vocal outputs in case of incorrect operation due to the wrong position of the sensors, which could not be well adherent to the skin. It can also transmit signals in real-time to a tablet or a PC using Bluetooth 2.0 or 5.0 protocol to allow experts to monitor signals or just upload data to a cloud server. In detail, the Sleep Profiler implements automatic event detection algorithms to determine an overall supine and non-supine AHI and ODI (Oxygen Desaturation Indices) [65]. The airflow detection rules were created to mimic how humans would identify apneas based on a >90% drop in airflow for 10 or more seconds and hypopneas based on a >30% reduction in airflow volume. The algorithms used sliding scale thresholds based on the strength of the airflow signal to reduce the identification of over-scored events when there is little airflow. With 3% SpO_2_ desaturation happening between 5 and 120 s from baseline and 1% recovery occurring within 30 s of the nadir, desaturation events required to validate hypopneas were identified using algorithms applied to the least filtered beat-by-beat SpO_2_ signal (four-beat fast average). Separate counts of desaturation occurrences were made.

In [66], tests were performed to understand the effectiveness of the Sleep Profiler headband; it was used to acquire 3 EEG signals, sampled at 256 Hz with a ± 1000 µV dynamic range and filtered with 0.1 Hz high-pass and 80 Hz low-pass filters. The detected signals were then evaluated by some experts, who analyzed the waveforms of the EEG signal to confirm detection veracity, especially near transitions from one stage to another. In summarizing, the five experts had a mean interscorer agreement of 75.9%, with 90.1%, 51.3%, 75.5%, 67.2%, and 91.1% for stages awake, N1, N2, N3, and REM stages, respectively; the mean kappa score was 0.70 (range 0.61 to 0.78) across all 10 comparisons. As a result, the performed studies have shown that Sleep Profiler is quite accurate. Even one night of recording is enough to characterize abnormal slow-wave sleep, spindle activity, and irregularities in heart rate.

Finally, Table 2 compares the commercial devices previously discussed from the point of view of the number and typology of detected parameters, integrated sensors, available biofeedbacks or post-interventions to improve sleep, and the cost to establish the best solution for tracking sleep. In conclusion, Sleep Profiler is the device that includes the largest number of sensors that track numerous physiological parameters despite its small size and lightness. The only flaw is that after 50 nights, the sensor strip has to be replaced and, probably, the headband too. Nevertheless, Sleep Profiler uses common Ag/AgCl disposable electrodes, which ensures a larger signal quality but requires their substitution after each use.

## 5. A Survey about Algorithms for Sleep Staging and Disorders Detection

This section proposes conventional and representation learning algorithms for sleep staging and disorder detection by analyzing the features extracted from biosignals acquirable through wearable devices. The aim is to provide some details about software tools commonly used for the abovementioned purposes, not lingering on acquisition modality according to the application scenario. In particular, Section 5.1 analyzes different algorithms for sleep stage classification based on AASM (American Academy of Sleep Medicine) guidelines. In contrast, Section 5.2 describes the implementation of algorithms that allow feature extraction for the early identification and treatment of sleep disorders.

### 5.1. Overview of Algorithms for Sleep Staging

As already stated, sleep plays an essential role in human life, but regrettably, many subjects suffer from sleep disorders, such as difficulty in initiating and maintaining sleep, circadian rhythm irregularities, and movement and breathing disorders, with serious repercussions in everyday life as well as on the subjects’ health. In detail, 70 million U.S. adults have sleep disorders, where 48% report snoring, 37.9% unintentionally falling asleep during the day, and 4.7% nodding off or falling asleep while driving [68,69]. The most prevalent specific sleep disturbance is insomnia, which is reported by 10% of individuals with chronic insomnia and 30% of adults with short-term disorders; besides, 25 million adults show OSA (i.e., 9–21% of women and 24–31% of men). Therefore, a scalable evaluation of the sleep of healthy people and patients with disorders can improve their life quality from a physical, mental, financial, and social point of view [70]. It can be carried out using non-invasive devices that accurately take a series of measurements, including EEG, EMG, EOG, and PPG, and, exploiting robust and efficient machine learning algorithms, analyze sleep architecture to determine its stages.

Moreover, the amount of information contained in these signals can be increased by developing new approaches [71]. For example, sleep/wake dynamics analysis can be conducted using the HRV (heart rate variability); it represents the fluctuation of the cardiac contraction times between each beat and the next due to physiological factors or external stimuli. In the scientific literature, this index is increasingly assuming a fundamental role since it represents the state of well-being of physiological systems, including sleep. HRV reflects autonomic nervous system activity, highlighting a higher parasympathetic activity during the NREMS phase and a balance variation from parasympathetic predominance to sympathetic hyperactivity, featuring the REMS phase [72]. Therefore, it can also be used to classify the individual’s current state: when the subject is awake, the heart rate is higher, and the heart rhythm is more variable; whereas when the subject is asleep, the parasympathetic autonomic nervous system dominates, the sympathetic component decreases with sleep depth, the heart rate is lower and reaches its minimum value during deep sleep (NREM stage), when the heart rhythm stabilizes.

In [71], J. Malik et al., to quantify HRV, proposed using a CNN (convolutional neural network), which can manage an enormous amount of data and identify useful elements for classification (e.g., sleep stage classification). Therefore, the ECG signal, acquired by a common 32-channel polygraph, was split into 30 s epochs; before passing it through 5 convolution blocks, it is concatenated with the previous 4 min and 30 s and normalized by subtracting its median value. In each convolution block, a bias is added; the output is passed through a rectified linear unit (ReLU) activation function. Then, there are two dense layers of 20 nodes each, associated with other biases and ReLU activation functions, finally feeding a 2-node output layer. The softmax function is used to normalize the network’s output, which, if related to the “wake” node and greater than or equal to the output of the “sleep” node during a given epoch, demonstrated that the subject was awake. Using the four databases, the CNN performances in distinguishing between wake and sleep stages can be expressed in accuracy, sensitivity, specificity, Cohen’s kappa coefficient, and AUC values (Table 3). This algorithm could be easily applied to data gathered by a head-worn wearable device acquiring the ECG signal on the user’s head, as reported in [73].

HRV was also the object of study in [74], as it is a low-cost alternative to PSG for automated sleep stage classification. In particular, to model long-term sleep-related information, M. Radha et al. proposed the LSTM (long short-term memory) network, which exploits ECG signals to distinguish four sleep stages: wake, REM, N1/N2, and N3 stages (Table 4). The proposed model considers the dependence between the night duration and the expected sleep stages, modeling a rigid sleep architecture restricted to short-term patterns; it is also inappropriate for insomnia patients. Connecting two stacks of LSTM multiple layers in parallel makes it possible to identify the past and future sleep epochs.

Also, HRV data can be extracted with sensors different from ECG, PPG, and heart sound signals; for instance, in [75], H. Hwang et al. focus on a stages classification method based on PVDF (polyvinylidene fluoride) sensor. Some tests were conducted to evaluate the method’s performance involving 12 normal subjects and 13 subjects suffering from OSA. Information on breathing (such as the average frequency and the variability of respiratory rate, useful for defining the NREM and REM stages) and the patient’s body movement (useful for defining the state of wakefulness) was retrieved from the PVDF’s sensor signal, exploiting the decision rules algorithm based on the IF-THEN statement. The test demonstrated that the developed method could classify the sleep stages with a 70.9% accuracy and a 0.48 kappa score. Instead, in [76], a model based on epoch location in a sleep cycle was presented, adapting a priori probabilities over time for different sleep stages. They recorded respiratory effort signals (using RIP—respiratory inductance plethysmography sensor) of 685 healthy participants chosen from the Sleep Heart Health Study data set. They used an XGBoost algorithm, a decision tree-based machine learning algorithm ideal for small and medium-structured data. The developed method reached a 0.56 ± 0.12 Cohen’s kappa. Instead, in [77], 3D accelerometers and optical PPG sensors were employed to extract HRV features and motion and respiratory movements using a non-temporal model. To validate the linear discriminant algorithm (LDA) used in this study to estimate sleep stages, 60 healthy adults aged from 18 to 60 years were involved in one-night recordings. The algorithm was trained on a known set of annotated sleep data from a target population and then applied to new sleep records, thus constituting a machine learning system for staging sleep. For each 30 s epoch, the classifier generates a different label (i.e., ‘Wake’, ‘Light’, ‘Deep’, or ‘REM’). Some post-processing criteria were implemented to penalize improbable physiological patterns, resulting in further gains in classifier accuracy. For instance, the transition between wake and deep sleep needs intermediate steps; thus, a single isolated wake epoch is improbable during a period of deep sleep epochs. In this case, that label can be converted to a surrounding one. As a result, the model achieved a 69% accuracy and a 0.52 ± 0.14 Cohen’s kappa.

Moreover, some sleep classification algorithms were proposed in the scientific literature from variables extracted from the respiration signal, such as the respiratory variability (RV) data [78]. K. Aggarwal et al. proposed a new approach for sleep staging based on CPAP (continuous positive air pressure) nasal flow signals and on a framework that combines CNN (to detect high-level features from flow sensors) and RNN (recurrent neural network to extract the feature’s context) [79]. Moreover, the neural network exploits a CRF (chain-structured conditional random field) algorithm to represent sleep dynamics. The presented architecture is a variation of ResNet (residual neural network) used in computer vision and time-series analysis. The system provides for the recording of the flow signals sampled at 32 Hz; for each 30 s epoch, the architecture estimates the corresponding sleep stage label: W (wake), R (REM), L (light sleep), or D (deep sleep). The proposed architecture was tested on 400 subjects suffering from sleep apnea, recording nasal flow signals sampled at 32 Hz for 7.5 h; the experimental results demonstrated that the proposed classification system achieved a 0.57 Cohen’s kappa and 74.1% accuracy.

EEG is the primary data source to determine the user’s sleep stage while discussing sleep staging. EEG is a useful indicator of the sleep stage, given the variation of its characteristics in amplitude and frequency as a function of the astronaut’s sleep phase [80]. EEG signals of certain frequencies: sleep spindles (12–14 Hz), slow waves (0.5–4 Hz), alpha waves (8–12 Hz), and theta oscillations (4–8 Hz) are very important in distinguishing the different phases of sleep, which can be easily extracted through common frequency analysis.

In addition, A. Malafeev et al. tested several machine learning algorithms, RF (random forest), LSTM networks, and CNN-LSTM networks, trained on two data sets [81]. Additionally, researchers classified sleep stages based on features or raw data using a supervised technique in machine learning. Features considered by the first approach are spindles (12–14 Hz) and slow (0.5–4 Hz), alpha (8–12 Hz), and theta (4–8 Hz) waves analyzed in the frequency domain. For classification, researchers proposed two methods: RF (random forest), based on decision trees, where every node corresponds to a feature, or ANN (artificial neural network), composed of interconnected neurons. The second approach involves DNNs (deep neural networks), an ANN that can model complex systems and automatically learn features using CNNs. The researchers implemented the learning of temporal structures of sleep by applying an HMM (hidden Markov model) to the output of RF classification and an MF (matrix factorization) to the data or applying RNNs, which consider the temporal structure of the data, also using information about future epochs. Some tests estimated the algorithms’ performance based on Cohen’s kappa. In addition, the study introduced in [82] illustrates a sleep staging method based on EEG signals, which involves signal pre-processing, characteristic parameters detection, and sleep stage identification. The pre-processing operation aims to reduce high-frequency background noise, low-frequency baseline wander, and artifact disturbance, apply a low-pass filter, split signals into 30 s data, and remove baseline drift and artifacts. The method employs the DWT (discrete wavelet transform) for deriving features, choosing a 4 Hz decomposition frequency since the EEG signal’s power is mostly concentrated between 0 and 30 Hz. The TSVM (transfer support vector machine) algorithm is used for EEG signal classification to automatically detect the sleep stage. The proposed method was evaluated using 60 sets of physiological signal data from the NSRR (National Sleep Research Resource) library, involving 6441 healthy subjects. Experimental analysis shows that the TSVM algorithm reaches an average of 88.05 TPR (true positive rate), a 96.11 TNR (true negative rate), and a 75.10 precision indicator.

Also, in [83], EEG signals are used for automatic sleep stage classification. The researchers proposed the IMBEFs (improved model-based essence features) model extracted from LE (locality energy) and DSSMs (dual state space models), estimated on LSBs (low-level sub-bands) and HSBs (high-level sub-bands), which are the result of EEG signals decomposition through WPD (wavelet packet decomposition). Begged trees classifier was chosen for sleep stages identification, among others, such as linear and quadratic discriminant, quadratic SVM, fine KNN, and RUSBoosted trees, because it achieved an overall accuracy, calculated on different databases, higher than others (about 95.35%). The sleep classifier achieved a 92.04% overall accuracy using the S-EDF database and an 81.65% overall accuracy using the ISRUC3 database.

Numerous solutions could be applied to the sleep staging problem, performing joint analysis of several biosignals (EEG, ECG, HR, HRV, RR, SpO_2_, etc.), together with the movement of some regions of the face (eyes, chin) [84]. Again, the classification algorithms are typically based on ML (neural network, SVM—support vector machine), LDA (linear discriminant analysis), and decision rules algorithms [85,86].

R. Agarwal et al., presented a computer-assisted sleep staging (CASS) method to standardize the analysis of recordings; it is based on features segmentation and iterative self-organization methodologies to identify patterns and then correlate them to a stage’s label [87]. Researchers used a data set composed of EEG, EOG, and EMG signals sampled at 128 Hz. The 12 involved subjects were aged from 17 to 62 years, and some of them suffered from sleep disorders, such as narcolepsy or sleep apnea. The classification is based on specific features: signal amplitude, dominant rhythm, FWE, presence of spindles, ASI (Alpha-Slow-wave Index), TSI (Theta-Slow-wave Index), and presence of EMs (eye movements). Post-processing operation allows substitution of a label in the case of a clear wrong assignment. Afterward, the presence of alpha and spindle activity, ASI, EMs, and atony are also evaluated using a decision tree algorithm.

In [88], the authors proposed a sleep stage identification algorithm based on HRV and EDR (ECG-derived respiration) obtained by ECG signals. The proposed method performs a baseline wander removal and R-peaks detection (PanTompkins algorithm). In particular, EDR is the extraction of information about breathing from ECG. This operation is performed using the N-PCA (neural-principal component analysis) approach, decreasing data set dimensionality and removing redundancy. EDR extraction can be performed by analyzing morphological variation, R-peaks, T-peaks amplitude variations, and area under the QRS complex, assuming that breathing alters ECG signals due to electrodes’ movements during breathing and thoracic impedance variations. Features evaluation and determination are performed using the Kruskal–Wallis method and the mRMR (minimum redundancy maximum relevance) algorithm, used as a multivariate filter to decrease features’ dimensionality, remove redundancy, and rank features through their pertinence to the target [89,90]. For classification, they used a multi-class SVM classifier with RBF (radial basis function) to identify wake, REM, and NREM stages, analyzing EDR and HRV data both in time and frequency domains. A k-fold cross-validation approach was used for implemented method validation, with k equal to 10. Researchers used the MIT-BIH Polysomnographic database to evaluate the detection algorithm, choosing 18 records, consisting of ECG, EEG, breathing signals, and blood pressure signals, of healthy subjects and patients suffering from sleep disorders aged between 35 and 49 years. Signals were acquired with a 250 Hz sampling frequency and then segmented every 30 s. Experimental tests showed an 81.76% accuracy and a 92.35% specificity for wake vs. sleep (WS) classification and a 75.15% accuracy and an 81.12% specificity for wake, REM, and NREM (WRN) classification.

In [91], the authors compared some classifiers, such as Bayesian LDs (linear discriminants), HMMs, and CRF, for simultaneous classification of multiple sleep stages (in particular, wake, REM, N1, N2, and N3 stages) based on cardiorespiratory features, evaluating the possibility of using them to analyze sleep of patients with OSA. Then, Hamilton–Tompkins QRS detector was used to detect QRS complexes, each composed of 3 deflections in ECG waveforms. Then, both ECG and RIP signals’ features were identified through variable-length windows applied on each 30 s epoch, except for those contaminated by artifacts, and were normalized, obtaining zero mean and unit standard deviation. The Bayesian LD classifier exploits the Bayes’ rule to decrease error probability by selecting each epoch’s most probable sleep stage. The authors used a k-fold cross-validation approach to validate the three classifiers, with k equal to 10. The results demonstrated that the Bayesian LD classifier achieved a 0.34 Cohen’s kappa and 48.76% accuracy, the HMM classifier a 0.25 Cohen’s kappa and 42.92% accuracy, and the CRF classifier a 0.42 Cohen’s kappa and 61.10% accuracy in identifying the five sleep stages. Therefore, the CRF classifier performed better than the others.

In [92], the authors presented an automated approach for sleep stage identification based on single-lead ECG signals, from which HRV and EDR are extracted. Classification is performed using beat detection, CPC (cardiopulmonary coupling) in the time and frequency domains, a trained CNN, and an SVM algorithm. CPC was calculated through the coherence, the power in specific bands of the cross-spectral density between EDR and RSA (respiratory sinus arrhythmia) signals, and the HRV cross-spectrogram in 5 min window. After, CPC spectrograms were used by CNN to extract corresponding features and identify sleep stages automatically. To validate implemented method, the researchers used a k-fold cross-validation technique, with k equal to 10, and applied that to each database considered above. As a result, the algorithm achieved a 75.6 ± 9.0% average accuracy and a 0.54 Cohen’s kappa for the SLPDB database, a 65.6 ± 1.3% average accuracy, and a 0.31 Cohen’s kappa for the CinC2018tDB database, and a 65.9 ± 0.7% average accuracy and a 0.47 Cohen’s kappa for the SHHSv1 database.

Finally, Table 5 summarizes the algorithms for sleep staging, classifying them according to the number and typology of detected parameters, algorithms involved in the proposed solution, and the number of participants involved in the research.

### 5.2. Algorithms for Detecting Sleep Disorders

The importance of sleep is well known, but around 60 sleep-related disorders have been identified [93], including OSA, from which an ever-increasing number of people suffer (in 2013, around 200 million cases, especially middle-aged men [94,95], in 2020 reaching 1 billion cases [96]). In addition to OSA patients, the number of subjects suffering from other sleep disorders is increasing, representing a serious problem because these disorders enhance morbidity and mortality risks, especially in older adults [97,98]. Thus, it is vital to recognize and monitor episodes associated with these problems by analyzing biosignals, such as chest movements and ECG, EEG, and EMG signals, to diagnose and treat sleep disorders early and choose the most effective therapy. The data analysis can be easily and effectively carried out using machine learning and deep learning algorithms.

The study in [99] focuses on sleep apnea detection through single-lead ECG signals, using machine learning algorithms, 14 conventional ones and 19 deep learning ones, including LDA, QDA (quadratic discriminate analysis), LR (logistic regression), Gaussian naïve Bayes classifiers, Gaussian process, SVMs, K-nearest neighbor (KNN), DT (decision tree), ET (extra tree), RF, AdaBoost, GB (gradient boosting), MLP (multi-layer perceptron), MV (majority voting), convolutional networks, and DRNNs (deep recurrent neural networks). To evaluate these models’ performance, the researchers conducted tests on three data sets (training, validation, and test data sets). After R-peaks extraction (pre-processing), the conventional machine learning approach included feature extraction: the time-domain, frequency-domain, and non-linear features were detected from the ECG data. After extraction, features were selected, discarding irrelevant ones, and data were dimensionally reduced by applying the Karhunen-Loève transform. Finally, the last step included classification through several machine learning algorithms (implemented in Python using Google Colaboratory Environment) to detect sleep apnea episodes. To train and test the proposed system, researchers used the k-fold cross-validation method, with k equal to 5. The efficiency of each feature in representing the onset of apnea episodes was measured and compared with that of other ones using the ET algorithm. The results indicated that the power of the ECG HF component (high frequency, from 0.15 to 0.4 Hz) achieved a higher score. Instead, deep learning algorithms performed even better because they automatically extract features from signals: the ZFNet-BiLSTM, ZFNet-GRU (gated recurrent unit), and VGG16-LSTM achieved the highest accuracy and specificity (88.13% and 92.27%), the highest sensitivity (84.26%), and the highest F-score (84.24%), respectively.

In [100], the authors proposed a method based on single-channel EEG signals for sleep disorders detection, namely insomnia, NFLE (nocturnal frontal lobe epilepsy), narcolepsy, RBD (REM behavior disorder), PLM (periodic leg movement) disorder, and SDB (sleep-disordered breathing). The researchers used CAP (cyclic alternating pattern) sleep database for the model’s creation, where sleep stages were classified as W (Wake), S1, S2, S3, S4, or REM. Afterward, the authors readapted them following the AASM criterion, thus combining S3 and S4 in the N3 deep sleep stage and naming S1 and S2 as N1 and N2 light sleep stages, respectively. Each sleep stage’s analysis contributed to the identification of sleep disorders. After acquiring raw EEG data, they were pre-processed for background noise removal (using a pass-band filter with finite impulse response and Kaiser window) and segmented into 30 s epochs. These lasts were classified into sleep stages using the labels defined by the database and AASM guidelines. Subsequently, EEG signals were filtered through biorthogonal THFB (triplet half-band filter bank) for epochs’ partition into eight sub-bands, from which Hjorth parameters (activity, mobility, and complexity) were extracted. Then, they are fed to different supervised machine learning algorithms for sleep disorders classification, namely EBT (ensemble bagged trees), EBooT (ensemble boosted trees), SVMs, and KNN. Among them, EBT and EBooT classifiers showed better performances. All tests and training were performed through MATLAB R2020a, using Statistics and Machine Learning Toolboxes, and the model was validated using k-fold cross-validation, with k equal to 10. The model using the EBT classifiers achieved a 99.23%, 96.17%, 98.21%, 98.82%, 98.30%, and 98.83% accuracy in detecting insomnia, NFLE, narcolepsy, RBD, PLM, and SDB, respectively; the model based on EbooT classifier achieved 98.6%, 95.2%, 97.3%, 99.1%, 98.4%, and 98.0% in detecting insomnia, NFLE, narcolepsy, RBD, PLM, and SDB, respectively.

The respiratory signal also represents a rich source of information regarding the patient’s physiological state, allowing the detection of breathing irregularities, such as sleep disorders (apnea, hypopnea, etc.). In addition, this signal can be easily extracted by processing other biosignals, which can be acquired through wearable devices. In detail, ECG and airflow signals can be used for apnea detection, with this last measured by a pulse oximeter [101]. Abnormalities in airflow induce a reduction in blood oxygen saturation level (SpO_2_), which can be exploited to automatically detect apnea episodes during sleep. In [101], the authors used three databases (HuGCDN2008, AED, and UCD) to create and train the model. The researchers proposed CNN to automatically detect sleep apnea episodes. It included many kinds of layers: three input layers that obtained raw data, convolutional kernels, which detected features, non-linear layers, which used ReLU, a pooling layer that increased training velocity by reducing data’s dimensionality, fully connected layers to link previous layer neuron and next layer one, batch normalization layers, normalized exponential function layer, and an output layer. Using a multi-objective hyperparameter algorithm (NSGA-II), CNN hyperparameters could be optimized and chosen without the involvement of an expert. This operation was performed on the database HuGCDN2008 and then tested to assess performances using the remaining two databases. Performances were evaluated and compared through a 2D ROC curve. The results indicated that the AED database achieved the highest accuracy (92.65%) in detecting sleep apnea from 1 min inputs.

In [102], the authors proposed a method based on HR and HRV’s spectral analysis to identify sleep stages and detect OSA episodes. Indeed, HRV’s normal values vary in the case of sleep disorders, whereas sleep staging relies on sympathetic and parasympathetic activity detection. In particular, the sympathovagal balance is assessed by the LF components, which cover the frequency range from 0.04 to 0.15 Hz; in contrast, the parasympathetic tone is determined by the HF components, which present frequencies greater than 0.15 Hz. Therefore, the balance between sympathetic and parasympathetic systems can be evaluated by LF/HF ratio. Experimental tests employed ECG recordings of both patients suffering from apnea and healthy subjects collected in the Apnea-ECG, MIT-BIH, St. Vincent’s, CHUS, and Fantasia databases.

Similarly, in [103], the authors presented a method that exploited a software package, called RHRV, for HRV analysis and chronic sleep disorders identification, implemented using the statistical environment R. At first, the algorithm extracted HR signals and detected the QRS complexes using the ecgpuwave function. In addition, an algorithm was developed for artifact identification and rejection. After that, a cubic spline interpolation with a 4 Hz frequency was applied to the signal to prepare it for power spectral analysis. The signal’s spectrogram was calculated using the STFT (Short-Time Fourier Transform) on a zero mean signal segment with a 30 s window and a 2.5 s displacement. After, the LF/HF ratios were calculated, obtaining on average: 1.29 for the Apnea-ECG database class A, 1.08 for Apnea-ECG database class C, 1.18 for the MIT-BIH database, 1.28 for St. Vincent’s database, 0.97 for CHUS database, and 1.06 for Fantasia database. As a result, Apnea-ECG database class A, MIT-BIH database, St. Vincent’s database, and CHUS database achieved a 90.0%, 77.78%, 52.00%, and 28.07% sensitivity, respectively.

In [104], different deep learning algorithms were designed for automatic sleep apnea detection from single-lead ECG biosignal. In their proposal, HRV and EDR were extracted from ECG signals, pre-processed, at first, to reduce artifacts and background noise, and then divided into 1 min segments. Afterward, using the Hamilton–Tompkins algorithm, R-peaks were detected, calculating their amplitude and R–R intervals through the Welch method, interpolated at 3 Hz. The derived signals are fed into deep learning algorithms, i.e., DRNNs (LSTM, GRU, and bidirectional LSTM-BiLSTM) and CNN (a variant of LeNet-5). PhysioNet Apnea-ECG Database v1.0.0 was used to train and test the developed models; it included 70 recordings from ECG signals digitized at 100 Hz. Subjects involved were aged 27 to 63 years and had a maximum of 86.8 AI (Apnea Index), 57.1 HI (Hypopnea Index), and 93.5 AHI. 90% of the data set was used for training, whereas the remaining 10% was for validation. As a result, LeNet achieved an 80.50% accuracy, 72.37% sensitivity, 85.31% specificity, and 73.97% F1-score; LSTM achieved an 80.18% accuracy, 75.06% sensitivity, 83.33% specificity, and 74.25% F1-score; GRU achieved an 80.10% accuracy, 77.22% sensitivity, 81.86% specificity, and 74.71% F1-score; BiLSTM achieved an 80.15% accuracy, 72.83% sensitivity, 84.65% specificity, and 73.65% F1-score. It is evident that CNN, particularly LeNet, achieved greater accuracy and specificity, which means that CNNs are more suitable for identifying sleep apnea episodes. However, GRU achieved the greatest sensitivity and F1 score, thus detecting fewer false positive negatives.

Table 6 summarizes the algorithms for detecting sleep disorders analyzed above, considering the number and typology of detected parameters, the used algorithms, and the extracted features.

According to us, the approach proposed in [102] offers the best perfective in detecting sleep detection systems since HRV can be easily and unobtrusively extracted using integrated PPG sensors without needing electrodes placed on the skin. In fact, the HRV can be extracted from the PPG signal collected by wearable devices [105], allowing discreet and accurate sleep stage detection.

## 6. Effects of COVID-19 Pandemic on Sleep

Poor quality of sleep, sleep deprivation, or sleep disorders constituted already a serious problem because they affected a large portion of the population, but recently they have considerably increased following the pandemic, even in subjects not affected by the COVID-19, due to increased anxiety, fear, depression, stress, and loss of daily routine [106,107]. During the pandemic, around 40% of the population had trouble sleeping (previously, 24% had sleep problems). Even new terms, “Coronasomnia” or “COVID-somnia”, have been coined to indicate the set of symptoms related to sleep disorders, including:

- Insomnia;
- Sleep disruptions;
- Irregularity of the circadian rhythm;
- Nightmares;
- Reduction in sleep quality and duration;
- Excessive daytime sleepiness;
- Decreased focus;
- Bad mood.

Moreover, these symptoms are common among frontline medical staff; they were already at a high risk of sleep deprivation because of their nighttime shifts. However, the pandemic further increased poor sleep quality rates, leading to the compromization of immune systems and declination of their cognitive performance, increasing the likelihood of becoming infected and not allowing them to do their job well. Moreover, a recent meta-analysis demonstrated that fear of contracting the virus, mandatory isolation and lockdown, job loss and financial problems, and all other restrictions caused higher rates of anxiety (from 6.3% to 50.9%), depression (from 14.6% to 48.3%), PTSD (post-traumatic stress disorder, from 7.0% to 53.8%), psychological distress (from 34.4% to 38.0%), and stress (from 8.1% to 81.9%). The effects of COVID-19 on daily life are evident, but there are also long-term ones, which researchers are still analyzing.

In [108], the authors conducted a meta-analysis using research data extracted from the following databases, scholarly search engines, and preprints servers: American Psychological Association PsycINFO, Cochrane, CINAHL (Cumulative Index to Nursing and Allied Health Literature), EBSCOhost, EMBASE, Google Scholar, MEDLINE, ProQuest Medical, ScienceDirect, Scopus, Web of Science, medRxiv.org, Preprints.org, psyarxiv.com, arXiv.org, and biorxiv.org. They examined issues related to the spreading of the COVID-19 pandemic, focusing on the sleep quantity and quality of patients affected by COVID-19, health care workers, and the population. Data extraction was systematically performed following the “Data extraction for complex meta-analysis (DECiMAL)” guide, which helps reviewers with complex meta-analysis, using Microsoft Excel or Microsoft Access for small data sets and STATA or R software for bigger ones [109]. The results are provided as a point estimate with 95% confidence intervals in a forest plot. Overall, 44 records were analyzed, involving 54231 subjects from 13 different nations. The investigation showed that China presented a pooled prevalence rate of sleep disorders of 26.5%, whereas Italy and France presented higher statistics (55.0% and 50.8%, respectively); Germany presented a 38.8% pooled prevalence rate. Analysis of the general population, health care workers, and patients showed a 32.3%, 36.0%, and 74.8% pooled prevalence rate, respectively. Furthermore, it turned out that the men, especially the elderly and those affected by COVID-19, were related to a higher prevalence of sleep disorders. Usually, studies on this topic estimated sleep disorders with PSQI (Pittsburg Sleep Quality Index); in particular, general population and health care workers achieved a 37.9% and a 39.7% PSQI, respectively. In this case, younger subject presented a higher PSQI. Another method used for estimating sleep disorders was ISI (Insomnia Severity Index); the general population presented a 29.7% prevalence rate.

In [110], the authors used wearable devices as a chance to monitor COVID-19 patients at home. In particular, they involved a mild COVID-19 patient, aged 53 years, who already used to monitor his sleep with wearable devices to analyze physiological alterations. In addition, he was under observation for 55 days through 3 wearable devices: Oura ring Gen 2, which continually monitored sleep, and Fitbit Versa 2 and iSleep Watch, which worked for 12 h a day. This study has shown that COVID-19 impacted sleep patterns (e.g., TST—total sleep time or WASO—wake after sleep onset) and that sleep disorders could influence disease development. Thus, sleep could be used as a biomarker to track the disease’s evolution. Sleep length gradually increased from 10 to 12 h each day. On the tenth day, the patient presented lymphopenia, a reduced level of lymphocytes in the blood whose function is to determine the immune response to external agents; this blood deficiency was also linked to poor sleep. In the next days, the results indicated a significant increase in sleep fragmentation, and the WASO cumulative duration increased (52 ± 12 versus 80 ± 12 min, *p* < 0.001); but on the nineteenth day, some improvements were recorded, and TST decreased returning to 7 h. Finally, sleep quality was enhanced two days before taste and smell returned to normal.

In [111], the researchers focused on the effects of backlit screen exposure just before sleep on sleep disorders during the mandatory isolation term (from the end of March to the end of April 2020) due to the COVID-19 pandemic. For this purpose, they involved 7107 subjects in a survey based on sleep quality according to PSQI, ISI, and a partial version of the MEQr (Morningness-Eveningness Questionnaire). A second survey involved 2123 subjects aged 18 to 82 years to investigate their habits regarding the use of backlit screens during the two hours before falling asleep and, in particular, if electronic device usage has increased, reduced, or remained unchanged during the restriction period. The study showed that subjects who use screens more tended to present lower sleep quality and sleep disorders, such as alterations in the sleep/wake cycle, moderate and severe insomnia, a reduced TST, difficulty falling asleep, and delayed bedtime and rising time. In this subjects’ group, the number of poor sleepers decreased by 10.7% (χ^2^ = 19.90, *p* = 0.04, and Cohen’s g = 0.18), and insomnia occurred 7.3% fewer times (χ^2^ = 12.21, *p* = 0.04, and Cohen’s g = 0.24). Finally, subjects who did not change habits showed changes from the point of view of insomnia occurrence, which, indeed, has decreased by 2.8% (χ^2^ = 188.51, *p* = 0.002, and Cohen’s g = 0.13) and poor sleepers’ number, that decreased by 2.3% (χ^2^ = 200.25, *p* = 0.17, and Cohen’s g = 0.04). The results suggested a direct link between evening backlit screen exposition and the temporal course of sleep disorders during isolation. In particular, longer screen exposure before falling asleep negatively influenced sleep patterns, increasing electronic device usage after sunset.

COVID-19 had a significant impact on the younger population; in [93], some studies conducted on children aged 12 years or under are summarized to analyze the disease’s effects on their sleep and determine eventual sleep disorders. School closures due to the pandemic led to alterations in children’s sleep patterns. Different works demonstrated that poor sleep quality has detrimental consequences on their social, emotional, cognitive, and functional development [112,113]; as a result, it affects children’s health and behavior. For their study, the researchers used different tools, such as SDSC (Sleep Disturbance Scale for Children), which quantifies sleep problems, PSQI (which indicates sleep quality, latency, duration, efficiency, and disorders), BISQ (Brief Infant Sleep Questionnaire), and CSHQ (Children’s Sleep Habits Questionnaire). The most researched metric in children was sleep duration, as in the pre-confinement literature [114]. Most children slept 9–10 h, consistent with earlier findings [115,116]. According to another study, younger children slept for longer periods, which is consistent with the results of other authors [117]. This conclusion, however, contradicted findings from other research, in which most children slept 8–10 h, with the shortest time occurring among the eldest children aged from 8 to 12 years [118,119].

A large-scale international survey was presented in [120]; the authors used a standardized questionnaire to investigate the sleep and day problems that occur before and after the COVID-19 pandemic. The study involved 22151 participants who answered questions about the effects of COVID-19, confinement, and financial problems on sleep. In detail, they used a linear regression model to adjust the results based on sex, age, ethnicity, educational level, previous sleep disturbance, and severity of the COVID-19 pandemic in the area where the questionnaire was presented.

The results indicated that during the outbreak, each daytime and sleep problem worsened by at least 10%. Some individuals have also reported improvements in daytime efficiency and sleep quality. For example, about 20% of individuals experienced a worsening sleep quality, while only 5% experienced an improvement. Poor sleep, early awakening, and daytime fatigue were also strongly linked to COVID-19. Poor sleep quality, problems falling asleep, and a decline in hypnotic use were all linked to confinement. Even in fully fitted logistic regression models, financial distress was linked to every sleep and daytime problem, including nightmares and exhaustion.

Moreover, electronic device usage increased among children following the confinement, which could affect sleep; a rise in latency time has been reported at all ages, by as much as 1 h [121,122,123]. Finally, the lack of routines and timetables during isolation severely impacted children’s behavior and sleep habits. The most significant alterations have been longer sleep durations, later bedtimes and waking up times, increased sleep latency, daytime drowsiness, and other sleep disorders.

In Ref. [124], consumer wearable devices were used to monitor different physiological parameters for detecting the onset of COVID-19 infectious disease. As for the effects on sleep, the obtained results have shown that the COVID-19 illness considerably alters steps and sleep patterns, with a significant increase in sleep duration. In Ref. [125], the authors developed a smartphone app that collects and processes the smartwatch and activity tracker data together with self-reported symptoms and COVID-19 diagnostic testing results. Similarly, they found that the sleep and activity of COVID-19-positive participants were impacted significantly more than COVID-19-negative ones, with an increase in the sleep duration of the COVID-19 symptomatic patients.

## 7. Conclusions

Sleep is a fundamental process that helps your body and mind to recover so that you may wake up feeling rejuvenated and aware. Healthy sleep is also important for keeping the body disease-free and healthy. The brain cannot operate correctly and efficiently without enough sleep. Your capacity to focus, think clearly, and process memory could be hampered by a lack of sleep or sleep-related disorders. For these reasons, sleep monitoring is increasingly interesting to the scientific community leading to the development of several systems and algorithms for tracking sleep and early detecting sleep disorders/dysfunctions. In this field, wearable devices are assuming a fundamental role, exploiting their ability to gather user biophysical signals accurately and uninvasively. This review aims to analyze the state-of-art of current wearable technologies and algorithms for monitoring sleep, aiming to stage and detect issues related to the sleep process (e.g., insomnia, restless legs syndrome, narcolepsy, and sleep apnea). In detail, a discussion about the importance of sleep monitoring is reported, pointing out physiological, psychological, and behavioral consequences of sleep deprivation. Then, an overview of wearable devices for sleep monitoring present in the scientific literature is introduced, lingering on architectural and functional aspects. Furthermore, several commercial wearable devices for monitoring sleep are discussed and analyzed, allowing a simplified and discreet detection of sleep stages and disorders directly at the user’s home. At the end of both overviews, comparative analyzes between the devices discussed are presented from a structural and functional point of view. Then, state-of-art about recent and advanced algorithms for sleep staging and detecting sleep disorders are introduced, starting from biosignals such as ECG, EEG, RR, EMG, and EOG. In addition, in this case, comparisons and technical evaluations of discussed algorithms are reported to highlight their potentialities and future prospects. Finally, scientific studies related to the effects on human life of the COVID-19 pandemic are discussed due to both the consequences of infections and changes in lifestyle.

The presented review focuses on finite wearable devices for sleep monitoring and algorithms for sleep staging and detecting sleep disorders, unlike other scientific works, which deal in a general and indistinct way with sensing techniques for sleep tracking, without distinguishing whether wearable systems and algorithms [14,19]. On the contrary, other reviews point out commercial wearable devices but do not consider prototype devices reported in the scientific literature and do not linger on algorithms for sleep classification and detecting sleep dysfunctions [13,126]. In addition, the presented work covers a relatively novel topic, treating the effect of the COVID-19 pandemic on the human body and lifestyle. In addition, an in-depth discussion on algorithms for detecting sleep disorders is presented, unlike other works considering only solutions for sleep classification [127]. Instead, in [128], the authors do not linger on algorithms and software tools but only on wearable devices reported in the literature, not considering commercial sensing systems.

## Figures and Tables

**Table 1:** Comparison among the scientific works previously discussed from the point of view of number and typology of detected parameters, availability of wireless connectivity to transfer data, and invasiveness.

Work	Number of Detected Parameters	Type of Detected Parameters	Availability of Wireless Module to Transfer Data	Tested Individuals	Accuracy	Sensitivity	Invasiveness
Mask B [39]	1	Eye movement	No	4	N.A. ^1^	N.A. ^1^	Low
Phymask [41]	5	Brain activity, eye movement, heart, and respiration rate, sleep stages, body movement	Yes	10	>0.8 ^2^	>0.8 ^2^	Low
HealthSOS [42]	1	Brain activity	Yes	37	92%	98%	Low
Smart Sleep Mask [43]	4	Eye movement, head position, temperature, and breathing sounds	Yes	1	N.A. ^1^	N.A. ^1^	Low
Chesma [44]	2	Eye movement, heart rate	Yes	1	N.A. ^1^	N.A. ^1^	Low
ARAM [45]	2	Respiration activity, body movement	No	6	N.A. ^1^	N.A. ^1^	Medium
Morfea [46]	3	Apnea and hypopnea events, chest movements, head position	Yes	1	93%	89%	Medium

^1^ Not available, ^2^ Cohen kappa coefficient.

**Table 2:** Comparison between the devices discussed above from the point of view of number and typology of the integrated sensors, gathered parameters, and cost.

Device	Number of Parameters Detected	Integrated Sensors	Gathered Parameters	Feedbacks/ Interventions	Cost
BrainBit [50]	4	EEG, PPG, EMG, EOG	Brain activity, heart rate, body movement, eye movement	Psychology and cognitive remediation	USD $499
SmartSleep [67]	1	EEG	Brain activity	Audio tones to boost the slow wave	USD $399
Muse S [53]	4	EEG, PPG, gyroscope, accelerometer	Brain activity, heart rate, breath rate, body movements	Digital sleeping pills (sleep stories and meditation, ambient soundscape, nature and music biofeedbacks)	USD $399
Dreem 2 [55]	4	EEG, PPG, gyroscope, accelerometer	Brain activity, heart rate, breath rate, body movement	CBT-I exercises	N.A. ^1^
iBand+ [58]	2	EEG, accelerometer, gyroscope	Brain activity, head movement	Audio tones to induce sleep	USD $449
Neuroon Open [59]	4	EEG, EOG, PPG, thermometer,	Brain activity, eye movement, body temperature, blood oxygenation	Audio tones to induce sleep	N.A. ^1^
Somni [60]	2	EOG, accelerometer	Eye movement, head movement	Audiovisual feedback to induce sleep	N.A. ^1^
BrainLink Pro [61]	4	EEG, PPG, gyroscope thermometer, accelerometer,	Brain activity, heart rate, body temperature, head movement	No	USD $259
Sleep Shepherd [62]	2	EEG, gyroscope, movement sensor	Brain activity head movement	Binaural tones to induce sleep	N.A. ^1^
Sleep Profiler [64]	5	EEG, EOG, EMG, accelerometer, ECG (optional), PPG (optional), nasal transducer (model SP29), pulse rate sensor (model SP29), oximeter (model SP29)	Brain activity, eye movement, head position, heart rate, quantitative snoring	No	N.A. ^1^

^1^ Not available.

**Table 3:** CNN model performance in distinguishing between wake and sleep stages, measured by true positive (TP), false positive (FP), false negative (FN), accuracy (ACC), area under the ROC curve (AUC), precision (PR), sensitivity (SE), and specificity (SP) [71].

	CGMH-Training	CGMH-Validation	DRAMS Subjects	UCDSADB
TP	4.464	1.800	1.777	1.838
FP	2.143	1.763	2.151	2.853
TN	31.550	14.906	14.532	12.883
FN	3.315	1.633	1.572	2.400
SE (%)	57.4	52.4	53.1	43.4
SP (%)	93.6	89.4	87.1	81.9
ACC (%)	86.8	83.1	81.4	73.7
PR (%)	67.6	50.5	45.2	39.2
F1	0.62	0.51	0.49	0.41
AUC	0.90	0.83	0.81	0.72
Kappa	0.54	0.41	0.38	0.24

**Table 4:** Summarizing table with the performance of the sleep stage classification algorithm presented in [74].

Stage	Precision	Accuracy	Cohen’s Kappa
Wake	0.73 ± 0.20	0.90 ± 0.07	0.63 ± 0.19
REM	0.71 ± 0.22	0.92 ± 0.04	0.68 ± 0.22
N1/N2	0.80 ± 0.11	0.79 ± 0.08	0.56 ± 0.15
N3	0.62 ± 0.33	0.92 ± 0.04	0.53 ± 0.27

**Table 5:** Summarizing table of the algorithms for sleep staging discussed above from the points of view of number and typology of detected parameters, employed algorithms, and the number of participants.

Authors	Number of Detected Parameters	Detected Parameters	Number of Sleep Stage	Accuracy [%]	Used Algorithms	Participants
J. Malik et al. [71]	2	ECG and PPG (deriving HRV and IHR ^1^)	2	86.8	CNN	56 patients and 90 healthy subjects
M. Radha et al. [74]	4	ECG, EEG, EOG, EMG (deriving HRV)	4	N.A. ^ 2 ^	LSTM	97 patients and 195 healthy subjects
H. Hwang et al. [75]	2	Breathing activity and body movements from the PVDF sensor	2	70.9	Decision rules algorithm	13 patients and 12 healthy subjects
A. Tataraidze et al. [76]	1	Effort signals using RIP	4	N.A. ^ 2 ^	XGB, a decision tree-based algorithm	685 healthy subjects
Z. Beattie et al. [77]	2	Breathing activity and body movements from a 3D accelerometer and optical PPG (deriving HRV)	4	69.0	LDA	60 healthy subjects
K. Aggarwal et al. [79]	1	Breathing activity from CPAP	4	74.1	CRF	400 patients
A. Malafeev et al. [81]	4	EEG, EMG, ECG, and EOG	5	N.A. ^ 2 ^	RF, LSTM, CNN-LSTM	23 patients and 18 healthy subjects
W. Wen [82]	1	EEG	5	N.A. ^ 2 ^	SVM	6641 healthy subjects
H. Shen et al. [83]	1	EEG	4	92.0	Begged trees	Patients and healthy subjects from three databases
R. Agarwal et al. [87]	3	EEG, EOG, and EMG	6	N.A. ^ 2 ^	CASS	12 subjects, some of them suffering from sleep disorders
A. Rahimi et al. [88]	1	ECG (deriving HRV and EDR)	2	81.8	SVM	Not specified
P. Fonseca et al. [91]	2	ECG and RIP	5	61.1%	LD, HMM, CRF	231 subjects, some of them suffering from sleep disorders
Q. Li et al. [92]	1	ECG (deriving HRV, EDR, and RSA)	3	85.1%	CPC, CNN, SVM	7451 subjects

^1^ Instantaneous heart rate; ^2^ not available.

**Table 6:** Summarizing table of the algorithms for detecting sleep disorders discussed above, considering the number and typology of detected parameters, used algorithms, and extracted features.

Work	Number of Detected Parameters	Detected Parameters	Type of Used Algorithm	Detected Sleep Disorder	Features Extracted
M. Bahrami et al. [99]	1	ECG	LDA, QDA, LR, Gaussian naïve Bayes classifiers, Gaussian process, SVMs, KNN, DT, ET, RF, AdaBoost, GB, MLP, MV, convolutional networks, and DRNNs	Sleep apnea	From R–R intervals: minimum, range, median, mean, standard deviation, skewness, kurtosis, the standard deviation of successive differences between adjacent R–R intervals, root mean square of successive differences between normal heartbeats, VLF, LF, HF, cardiovagal index, cardio sympathetic index
S. S. Mostafa et al. [101]	1	EEG	EBT; EBooT, SVMs, and KNN	OSA	Activity, mobility, and complexity
M. Sharma et al. [100]	2	ECG and SpO_2_	CNN and NSGA-II	Insomnia, NFLE, RBD, PLM disorder, and SDB	N.A. ^1^
M. J. Lado et al. [102]	1	ECG (deriving HR and HRV)	RHRV	OSA	LF/HF quotient
M. Bahrami et al. [104]	1	ECG	DRNNs and CNN	Sleep apnea	R-peak amplitude and R–R intervals

^1^ Not available.

## Data Availability

Data of our study are available upon request.

## References

[1] Worley S.L. (2018). The Extraordinary Importance of Sleep. Pharm. Ther..

[2] Okano K., Kaczmarzyk J.R., Dave N., Gabrieli J.D.E., Grossman J.C. (2019). Sleep Quality, Duration, and Consistency Are Associated with Better Academic Performance in College Students. NPJ Sci. Learn..

[3] Kapsi S., Katsantoni S., Drigas A. (2020). The Role of Sleep and Impact on Brain and Learning. Int. J. Recent Contrib. Eng. Sci. IT (IJES).

[4] Garbarino S., Lanteri P., Bragazzi N.L., Magnavita N., Scoditti E. (2021). Role of Sleep Deprivation in Immune-Related Disease Risk and Outcomes. Commun. Biol..

[5] Gottlieb D.J., Ellenbogen J.M., Bianchi M.T., Czeisler C.A. (2018). Sleep Deficiency and Motor Vehicle Crash Risk in the General Population: A Prospective Cohort Study. BMC Med..

[6] Peng Z., Dai C., Ba Y., Zhang L., Shao Y., Tian J. (2020). Effect of Sleep Deprivation on the Working Memory-Related N2-P3 Components of the Event-Related Potential Waveform. Front. Neurosci..

[7] Colten H.R., Altevogt B.M., Institute of Medicine (US) Committee on Sleep Medicine and Research (2006). Sleep Disorders and Sleep Deprivation: An Unmet Public Health Problem.

[8] Kales A., Soldatos C.R., Kales J.D. (1987). Sleep Disorders: Insomnia, Sleepwalking, Night Terrors, Nightmares, and Enuresis. Ann. Intern. Med..

[9] Sack R.L., Auckley D., Auger R.R., Carskadon M.A., Wright K.P., Vitiello M.V., Zhdanova I.V. (2007). Circadian Rhythm Sleep Disorders: Part I, Basic Principles, Shift Work and Jet Lag Disorders. Sleep.

[10] Gigli G.L., Adorati M., Dolso P., Piani A., Valente M., Brotini S., Budai R. (2004). Restless Legs Syndrome in End-Stage Renal Disease. Sleep Med..

[11] MedlinePlus Sleep Disorders. https://medlineplus.gov/sleepdisorders.html.

[12] Kelly J.M., Strecker R.E., Bianchi M.T. (2012). Recent Developments in Home Sleep-Monitoring Devices. ISRN Neurol.

[13] Robbins R., Seixas A., Masters L.W., Chanko N., Diaby F., Vieira D., Jean-Louis G. (2019). Sleep Tracking: A Systematic Review of the Research Using Commercially Available Technology. Curr. Sleep Med. Rep..

[14] Hussain Z., Sheng Q.Z., Zhang W.E., Ortiz J., Pouriyeh S. (2022). A Review of the Non-Invasive Techniques for Monitoring Different Aspects of Sleep. ACM Trans. Comput. Healthc..

[15] Buysse D.J. (2014). Sleep Health: Can We Define It? Does It Matter?. Sleep.

[16] De Fazio R., De Vittorio M., Visconti P. (2022). A BLE-Connected Piezoresistive and Inertial Chest Band for Remote Monitoring of the Respiratory Activity by an Android Application: Hardware Design and Software Optimization. Future Internet.

[17] De Fazio R., Al-Hinnawi A.-R., De Vittorio M., Visconti P. (2022). An Energy-Autonomous Smart Shirt Employing Wearable Sensors for Users’ Safety and Protection in Hazardous Workplaces. Appl. Sci..

[18] De Fazio R., Stabile M., De Vittorio M., Velázquez R., Visconti P. (2021). An Overview of Wearable Piezoresistive and Inertial Sensors for Respiration Rate Monitoring. Electronics.

[19] Imtiaz S.A. (2021). A Systematic Review of Sensing Technologies for Wearable Sleep Staging. Sensors.

[20] Schutte-Rodin S., Deak M.C., Khosla S., Goldstein C.A., Yurcheshen M., Chiang A., Gault D., Kern J., O’Hearn D., Ryals S. (2021). Evaluating Consumer and Clinical Sleep Technologies: An American Academy of Sleep Medicine Update. J. Clin. Sleep Med..

[21] Chinoy E.D., Cuellar J.A., Jameson J.T., Markwald R.R. (2022). Performance of Four Commercial Wearable Sleep-Tracking Devices Tested under Unrestricted Conditions at Home in Healthy Young Adults. Nat. Sci. Sleep.

[22] Alimoradi Z., Broström A., Tsang H.W.H., Griffiths M.D., Haghayegh S., Ohayon M.M., Lin C.-Y., Pakpour A.H. (2021). Sleep Problems during COVID-19 Pandemic and Its’ Association to Psychological Distress: A Systematic Review and Meta-Analysis. eClinicalMedicine.

[23] Lin Y.N., Liu Z.R., Li S.Q., Li C.X., Zhang L., Li N., Sun X.W., Li H.P., Zhou J.P., Li Q.Y. (2021). Burden of Sleep Disturbance During COVID-19 Pandemic: A Systematic Review. Nat. Sci. Sleep.

[24] Islam M.K., Molla M.M.A., Hasan P., Sharif M.M., Hossain F.S., Amin M.R., Rahman M.R. (2022). Persistence of Sleep Disturbance among Post-COVID Patients: Findings from a 2-Month Follow-up Study in a Bangladeshi Cohort. J. Med. Virol..

[25] Tachiquin R., Velázquez R., Del-Valle-Soto C., Gutiérrez C.A., Carrasco M., De Fazio R., Trujillo-León A., Visconti P., Vidal-Verdú F. (2021). Wearable Urban Mobility Assistive Device for Visually Impaired Pedestrians Using a Smartphone and a Tactile-Foot Interface. Sensors.

[26] de Fazio R., Perrone E., Velázquez R., De Vittorio M., Visconti P. (2021). Development of a Self-Powered Piezo-Resistive Smart Insole Equipped with Low-Power BLE Connectivity for Remote Gait Monitoring. Sensors.

[27] Chokroverty S. (2010). Overview of Sleep & Sleep Disorders. Indian J. Med. Res..

[28] Thorpy M.J. (2012). Classification of Sleep Disorders. Neurotherapeutics.

[29] Surantha N., Kusuma G.P., Isa S.M. (2016). Internet of Things for Sleep Quality Monitoring System: A Survey. Proceedings of the 2016 11th International Conference on Knowledge, Information and Creativity Support Systems (KICSS).

[30] Wickboldt A.T., Bowen A.F., Kaye A.J., Kaye A.M., Bueno F.R., Kaye A.D. (2012). Sleep Physiology, Abnormal States, and Therapeutic Interventions. Ochsner J..

[31] Chesson A.L., Ferber R.A., Fry J.M., Grigg-Damberger M., Hartse K.M., Hurwitz T.D., Johnson S., Kader G.A., Littner M., Rosen G. (1997). The Indications for Polysomnography and Related Procedures. Sleep.

[32] Dijk D.-J., Neri D.F., Wyatt J.K., Ronda J.M., Riel E., Ritz-De Cecco A., Hughes R.J., Elliott A.R., Prisk G.K., West J.B. (2001). Sleep, Performance, Circadian Rhythms, and Light-Dark Cycles during Two Space Shuttle Flights. Am. J. Physiol.-Regul. Integr. Comp. Physiol..

[33] Oh C.-M., Kim H.Y., Na H.K., Cho K.H., Chu M.K. (2019). The Effect of Anxiety and Depression on Sleep Quality of Individuals with High Risk for Insomnia: A Population-Based Study. Front. Neurol..

[34] Silvani A., Dampney R.A.L. (2013). Central Control of Cardiovascular Function during Sleep. Am. J. Physiol.-Heart Circ. Physiol..

[35] Somers V.K., Dyken M.E., Mark A.L., Abboud F.M. (1993). Sympathetic-Nerve Activity during Sleep in Normal Subjects. N. Engl. J. Med..

[36] Murali N.S., Svatikova A., Somers V.K. (2003). Cardiovascular Physiology and Sleep. Front. Biosci..

[37] Anusha A.S., Preejith S.P., Akl T.J., Sivaprakasam M. (2022). Electrodermal Activity Based Autonomic Sleep Staging Using Wrist Wearable. Biomed. Signal Process. Control.

[38] Mayo Clinic Polysomnography (Sleep Study). https://www.mayoclinic.org/tests-procedures/polysomnography/about/pac-20394877.

[39] Beach C., Karim N., Casson A.J. A Graphene-Based Sleep Mask for Comfortable Wearable Eye Tracking. Proceedings of the 2019 41st Annual International Conference of the IEEE Engineering in Medicine and Biology Society (EMBC).

[40] Greco V., Bergamo D., Cuoccio P., Konkoly K.R., Lombardo K.M., Lewis P.A. (2022). Wearing an Eye Mask during Overnight Sleep Improves Episodic Learning and Alertness. bioRxiv-Neurosci..

[41] Rostaminia S., Homayounfar S.Z., Kiaghadi A., Andrew T.L., Ganesan D. (2010). PhyMask: Robust Sensing of Brain Activity and Physiological Signals during Sleep with an All-Textile Eye Mask. arXiv.

[42] Hussain I., Park S.J. (2020). HealthSOS: Real-Time Health Monitoring System for Stroke Prognostics. IEEE Access.

[43] Dang B., Dicarlo J., Lukashov S., Hinds N., Reinen J., Wen B., Hao T., Bilal E., Rogers J. Development of a Smart Sleep Mask with Multiple Sensors. Proceedings of the 2021 43rd Annual International Conference of the IEEE Engineering in Medicine Biology Society (EMBC).

[44] Homayounfar S.Z., Rostaminia S., Kiaghadi A., Chen X., Alexander E.T., Ganesan D., Andrew T.L. (2020). Multimodal Smart Eyewear for Longitudinal Eye Movement Tracking. Matter.

[45] Puri R.S., Athanassiadis A.G., Gill N., Sathya S.S., Rathod G., Wahi A., Satat G., Majmudar M., Shah P. (2016). Design and Preliminary Evaluation of a Wearable Device for Mass-Screening of Sleep Apnea. Annu. Int. Conf. IEEE Eng. Med. Biol. Soc..

[46] Manoni A., Loreti F., Radicioni V., Pellegrino D., Della Torre L., Gumiero A., Halicki D., Palange P., Irrera F. (2020). A New Wearable System for Home Sleep Apnea Testing, Screening, and Classification. Sensors.

[47] Kuo C.-E., Liang S.-F., Li Y.-C., Cherng F.-Y., Lin W.-C., Chen P.-Y., Liu Y.-C., Shaw F.-Z. An EOG-Based Sleep Monitoring System and Its Application on On-Line Sleep-Stage Sensitive Light Control. Proceedings of the International Conference on Physiological Computing Systems (PhyCS).

[48] Hei Y., Yuan T., Fan Z., Yang B., Hu J. (2022). Sleep Staging Classification Based on a New Parallel Fusion Method of Multiple Sources Signals. Physiol. Meas..

[49] Goldstein B., Sakharov V., Bulanov S. (2020). Personal Apparatus for Conducting Electroencephalography. U.S. Patent.

[50] Brainbit Brainbit Manual. http://brainbit.com/.

[51] Lopez-Gordo M.A., Sanchez-Morillo D., Valle F.P. (2014). Dry EEG Electrodes. Sensors.

[52] Diep C., Garcia-Molina G., Jasko J., Manousakis J., Ostrowski L., White D., Anderson C. (2021). Acoustic Enhancement of Slow Wave Sleep on Consecutive Nights Improves Alertness and Attention in Chronically Short Sleepers. Sleep Med..

[53] Muse™ Headband Manual. https://choosemuse.com/.

[54] Western University, Canada (2021). Assessing the Effects of the Muse Sleep Intervention on Sleep.

[55] Dreem|Sleep Pioneers. https://dreem.com/en/.

[56] da Costa T.D., Vara M.D.F.F., Cristino C.S., Zanella T.Z., Neto G.N.N., Nohama P. (2019). Breathing Monitoring and Pattern Recognition with Wearable Sensors.

[57] Arnal P.J., Thorey V., Debellemaniere E., Ballard M.E., Bou Hernandez A., Guillot A., Jourde H., Harris M., Guillard M., Van Beers P. (2020). The Dreem Headband Compared to Polysomnography for Electroencephalographic Signal Acquisition and Sleep Staging. Sleep.

[58] IBand+ EEG Headband|Sleep Improvement & Lucid Dreaming Wearable Device. https://www.ibandplus.com/.

[59] Neuroon Open: World’s Smartest Sleep Tracker. https://www.indiegogo.com/projects/2172509.

[60] Somni Mask—The Easiest Way to Lucid Dreaming. https://somni.org/.

[61] BrainLink by Macrotellect|Healthy Brainwaves for Everyone. http://www.macrotellect.com/.

[62] Sleep Shepherd: Sleep Optimizer and Tracker. https://sleepshepherd.com/.

[63] Kim Y., Moon J., Lee H.-J., Bae C.-S., Sohn S. Integration of Electroencephalography Based Services into Consumer Electronics. Proceedings of the 2012 IEEE 16th International Symposium on Consumer Electronics.

[64] Sleep Profiler™ Specifications. https://www.advancedbrainmonitoring.com/products/sleep-profiler#section-specification.

[65] Levendowski D.J., Hamilton G.S., St. Louis E.K., Penzel T., Dawson D., Westbrook P.R. (2019). A Comparison between Auto-Scored Apnea-Hypopnea Index and Oxygen Desaturation Index in the Characterization of Positional Obstructive Sleep Apnea. Nat. Sci. Sleep.

[66] Levendowski D.J., Ferini-Strambi L., Gamaldo C., Cetel M., Rosenberg R., Westbrook P.R. (2017). The Accuracy, Night-to-Night Variability, and Stability of Frontopolar Sleep Electroencephalography Biomarkers. J. Clin. Sleep Med..

[67] SmartSleep Deep Sleep Headband. https://www.usa.philips.com/c-e/smartsleep/deep-sleep-headband.html.

[68] Centers for Disease Control and Prevention (CDC) (2011). Unhealthy Sleep-Related Behaviors—12 States, 2009. MMWR Morb. Mortal. Wkly. Rep..

[69] American Sleep Association Sleep Statistics: Data about Sleep Disorders. https://www.sleepassociation.org/about-sleep/sleep-statistics/.

[70] Kang D.Y., DeYoung P.N., Malhotra A., Owens R.L., Coleman T.P. (2018). A State Space and Density Estimation Framework for Sleep Staging in Obstructive Sleep Apnea. IEEE Trans. Biomed. Eng..

[71] Malik J., Lo Y.-L., Wu H. (2018). Sleep-Wake Classification via Quantifying Heart Rate Variability by Convolutional Neural Network. Physiol. Meas..

[72] Chouchou F., Desseilles M. (2014). Heart Rate Variability: A Tool to Explore the Sleeping Brain?. Front. Neurosci..

[73] Gil B., Anastasova S., Yang G.Z. (2019). A Smart Wireless Ear-Worn Device for Cardiovascular and Sweat Parameter Monitoring during Physical Exercise: Design and Performance Results. Sensors.

[74] Radha M., Fonseca P., Moreau A., Ross M., Cerny A., Anderer P., Long X., Aarts R.M. (2019). Sleep Stage Classification from Heart-Rate Variability Using Long Short-Term Memory Neural Networks. Sci. Rep..

[75] Hwang S.H., Lee Y.J., Jeong D.U., Park K.S. (2016). Unconstrained Sleep Stage Estimation Based on Respiratory Dynamics and Body Movement. Methods Inf. Med..

[76] Tataraidze A., Anishchenko L., Korostovtseva L., Bochkarev M., Sviryaev Y., Ivashov S. (2017). Estimation of a Priori Probabilities of Sleep Stages: A Cycle-Based Approach. Annu. Int. Conf. IEEE Eng. Med. Biol. Soc..

[77] Beattie Z., Oyang Y., Statan A., Ghoreyshi A., Pantelopoulos A., Russell A., Heneghan C. (2017). Estimation of Sleep Stages in a Healthy Adult Population from Optical Plethysmography and Accelerometer Signals. Physiol. Meas..

[78] Yang J., Keller J.M., Popescu M., Skubic M. (2016). Sleep Stage Recognition Using Respiration Signal. Annu. Int. Conf. IEEE Eng. Med. Biol. Soc..

[79] Aggarwal K., Khadanga S., Joty S., Kazaglis L., Srivastava J. A Structured Learning Approach with Neural Conditional Random Fields for Sleep Staging. Proceedings of the 2018 IEEE International Conference on Big Data (Big Data).

[80] Purves D., Augustine G.J., Fitzpatrick D., Katz L.C., LaMantia A.-S., McNamara J.O., Williams S.M. (2001). Stages of Sleep. Neuroscience.

[81] Malafeev A., Laptev D., Bauer S., Omlin X., Wierzbicka A., Wichniak A., Jernajczyk W., Riener R., Buhmann J., Achermann P. (2018). Automatic Human Sleep Stage Scoring Using Deep Neural Networks. Front. Neurosci..

[82] Wen W. (2021). Sleep Quality Detection Based on EEG Signals Using Transfer Support Vector Machine Algorithm. Front. Neurosci..

[83] Shen H., Ran F., Xu M., Guez A., Li A., Guo A. (2020). An Automatic Sleep Stage Classification Algorithm Using Improved Model Based Essence Features. Sensors.

[84] Faust O., Razaghi H., Barika R., Ciaccio E.J., Acharya U.R. (2019). A Review of Automated Sleep Stage Scoring Based on Physiological Signals for the New Millennia. Comput. Methods Programs Biomed..

[85] Liang S.-F., Kuo C.-E., Hu Y.-H., Cheng Y.-S. (2011). A Rule-Based Automatic Sleep Staging Method. Annu. Int. Conf. IEEE Eng. Med. Biol. Soc..

[86] Tagluk M.E., Sezgin N., Akin M. (2010). Estimation of Sleep Stages by an Artificial Neural Network Employing EEG, EMG and EOG. J. Med. Syst..

[87] Agarwal R., Gotman J. (2001). Computer-Assisted Sleep Staging. IEEE Trans. Biomed. Eng..

[88] Rahimi A., Safari A., Mohebbi M. Sleep Stage Classification Based on ECG-Derived Respiration and Heart Rate Variability of Single-Lead ECG Signal. Proceedings of the 2019 26th National and 4th International Iranian Conference on Biomedical Engineering (ICBME).

[89] McKight P.E., Najab J. (2010). Kruskal-Wallis Test. The Corsini Encyclopedia of Psychology.

[90] Ramírez-Gallego S., Lastra I., Martínez-Rego D., Bolón-Canedo V., Benítez J.M., Herrera F., Alonso-Betanzos A. (2017). Fast-MRMR: Fast Minimum Redundancy Maximum Relevance Algorithm for High-Dimensional Big Data. Int. J. Intell. Syst..

[91] Fonseca P., den Teuling N., Long X., Aarts R.M. (2018). A Comparison of Probabilistic Classifiers for Sleep Stage Classification. Physiol. Meas..

[92] Li Q., Li Q., Liu C., Shashikumar S.P., Nemati S., Clifford G.D. (2018). Deep Learning in the Cross-Time Frequency Domain for Sleep Staging from a Single-Lead Electrocardiogram. Physiol. Meas..

[93] Sateia M.J. (2014). International Classification of Sleep Disorders-Third Edition. Chest.

[94] Young T., Palta M., Dempsey J., Skatrud J., Weber S., Badr S. (1993). The Occurrence of Sleep-Disordered Breathing among Middle-Aged Adults. N. Engl. J. Med..

[95] Zhang J., Zhang Q., Wang Y., Qiu C. A Real-Time Auto-Adjustable Smart Pillow System for Sleep Apnea Detection and Treatment. Proceedings of the 2013 ACM/IEEE International Conference on Information Processing in Sensor Networks (IPSN).

[96] Lyons M.M., Bhatt N.Y., Pack A.I., Magalang U.J. (2020). Global Burden of Sleep-Disordered Breathing and Its Implications. Respirology.

[97] Acquavella J., Mehra R., Bron M., Suomi J.M.-H., Hess G.P. (2020). Prevalence of Narcolepsy and Other Sleep Disorders and Frequency of Diagnostic Tests from 2013–2016 in Insured Patients Actively Seeking Care. J. Clin. Sleep Med..

[98] Neikrug A.B., Ancoli-Israel S. (2010). Sleep Disorders in the Older Adult—A Mini-Review. Gerontology.

[99] Bahrami M., Forouzanfar M. (2022). Sleep Apnea Detection from Single-Lead ECG: A Comprehensive Analysis of Machine Learning and Deep Learning Algorithms. IEEE Trans. Instrum. Meas..

[100] Sharma M., Tiwari J., Patel V., Acharya U.R. (2021). Automated Identification of Sleep Disorder Types Using Triplet Half-Band Filter and Ensemble Machine Learning Techniques with EEG Signals. Electronics.

[101] Mostafa S.S., Mendonça F., Ravelo-Garcia A.G., Gabriel Juliá-Serdá G., Morgado-Dias F. (2020). Multi-Objective Hyperparameter Optimization of Convolutional Neural Network for Obstructive Sleep Apnea Detection. IEEE Access.

[102] Lado M.J., Vila X.A., Rodríguez-Liñares L., Méndez A.J., Olivieri D.N., Félix P. (2011). Detecting Sleep Apnea by Heart Rate Variability Analysis: Assessing the Validity of Databases and Algorithms. J. Med. Syst..

[103] Vila X.A., Lado M.J., Mendez A.J., Olivieri D.N., Linares L.R. An R Package for Heart Rate Variability Analysis. Proceedings of the 2009 IEEE International Symposium on Intelligent Signal Processing.

[104] Bahrami M., Forouzanfar M. Detection of Sleep Apnea from Single-Lead ECG: Comparison of Deep Learning Algorithms. Proceedings of the 2021 IEEE International Symposium on Medical Measurements and Applications (MeMeA).

[105] Alqaraawi A., Alwosheel A., Alasaad A. (2016). Heart Rate Variability Estimation in Photoplethysmography Signals Using Bayesian Learning Approach. Healthc. Technol. Lett..

[106] Bhat S., Chokroverty S. (2021). Sleep Disorders and COVID-19. Sleep Med..

[107] Coronasomnia: Definition, Symptoms, and Solutions. https://www.sleepfoundation.org/covid-19-and-sleep/coronasomnia.

[108] Jahrami H., BaHammam A.S., Bragazzi N.L., Saif Z., Faris M., Vitiello M.V. (2021). Sleep Problems during the COVID-19 Pandemic by Population: A Systematic Review and Meta-Analysis. J. Clin. Sleep Med..

[109] Pedder H., Sarri G., Keeney E., Nunes V., Dias S. (2016). Data Extraction for Complex Meta-Analysis (DECiMAL) Guide. Syst. Rev..

[110] Metlaine A., Sauvet F., Chennaoui M., Leger D., Elbaz M. (2021). Sleep and COVID-19. A Case Report of a Mild COVID-19 Patient Monitored by Consumer-Targeted Sleep Wearables. Sensors.

[111] Salfi F., Amicucci G., Corigliano D., D’Atri A., Viselli L., Tempesta D., Ferrara M. (2021). Changes of Evening Exposure to Electronic Devices during the COVID-19 Lockdown Affect the Time Course of Sleep Disturbances. Sleep.

[112] Lim M.T.C., Ramamurthy M.B., Aishworiya R., Rajgor D.D., Tran A.P., Hiriyur P., Kunaseelan S., Jabri M., Goh D.Y.T. (2021). School Closure during the Coronavirus Disease 2019 (COVID-19) Pandemic—Impact on Children’s Sleep. Sleep Med..

[113] Casagrande M., Favieri F., Tambelli R., Forte G. (2020). The Enemy Who Sealed the World: Effects Quarantine Due to the COVID-19 on Sleep Quality, Anxiety, and Psychological Distress in the Italian Population. Sleep Med..

[114] Sun W., Ling J., Zhu X., Lee T.M.-C., Li S.X. (2019). Associations of Weekday-to-Weekend Sleep Differences with Academic Performance and Health-Related Outcomes in School-Age Children and Youths. Sleep Med. Rev..

[115] Cachón-Zagalaz J., Zagalaz-Sánchez M.L., Arufe-Giráldez V., Sanmiguel-Rodríguez A., González-Valero G. (2021). Physical Activity and Daily Routine among Children Aged 0–12 during the COVID-19 Pandemic in Spain. Int. J. Environ. Res. Public Health.

[116] Zhao Y., Guo Y., Xiao Y., Zhu R., Sun W., Huang W., Liang D., Tang L., Zhang F., Zhu D. (2020). The Effects of Online Homeschooling on Children, Parents, and Teachers of Grades 1–9 during the COVID-19 Pandemic. Med. Sci. Monit..

[117] Aguilar-Farias N., Toledo-Vargas M., Miranda-Marquez S., Cortinez-O’Ryan A., Cristi-Montero C., Rodriguez-Rodriguez F., Martino-Fuentealba P., Okely A.D., del Pozo Cruz B. (2021). Sociodemographic Predictors of Changes in Physical Activity, Screen Time, and Sleep among Toddlers and Preschoolers in Chile during the COVID-19 Pandemic. Int. J. Environ. Res. Public Health.

[118] Dutta K., Mukherjee R., Sen D., Sahu S. (2022). Effect of COVID-19 Lockdown on Sleep Behavior and Screen Exposure Time: An Observational Study among Indian School Children. Biol. Rhythm. Res..

[119] Abid R., Ammar A., Maaloul R., Souissi N., Hammouda O. (2021). Effect of COVID-19-Related Home Confinement on Sleep Quality, Screen Time and Physical Activity in Tunisian Boys and Girls: A Survey. Int. J. Environ. Res. Public Health.

[120] Partinen M., Holzinger B., Morin C.M., Espie C., Chung F., Penzel T., Benedict C., Bolstad C.J., Cedernaes J., Chan R.N.Y. (2021). Sleep and Daytime Problems during the COVID-19 Pandemic and Effects of Coronavirus Infection, Confinement and Financial Suffering: A Multinational Survey Using a Harmonised Questionnaire. BMJ Open.

[121] Hale L., Guan S. (2015). Screen Time and Sleep among School-Aged Children and Adolescents: A Systematic Literature Review. Sleep Med. Rev..

[122] Adıbelli D., Sümen A. (2020). The Effect of the Coronavirus (COVID-19) Pandemic on Health-Related Quality of Life in Children. Child. Youth Serv. Rev..

[123] Liu Z., Tang H., Jin Q., Wang G., Yang Z., Chen H., Yan H., Rao W., Owens J. (2021). Sleep of Preschoolers during the Coronavirus Disease 2019 (COVID-19) Outbreak. J. Sleep Res..

[124] Mishra T., Wang M., Metwally A.A., Bogu G.K., Brooks A.W., Bahmani A., Alavi A., Celli A., Higgs E., Dagan-Rosenfeld O. (2020). Pre-symptomatic detection of COVID-19 from smartwatch data. Nat. Biomed. Eng..

[125] Quer G., Radin J.M., Gadaleta M., Baca-Motes K., Ariniello L., Ramos E., Kheterpal V., Topol E.J., Steinhubl S.R. (2020). Wearable sensor data and self-reported symptoms for COVID-19 detection. Nat. Med..

[126] Chi J., Cao W., Gu Y. (2020). Recent Progress in Sleep Quality Monitoring and Non-Drug Sleep Improvement. Front. Hum. Neurosci..

[127] Roomkham S., Lovell D., Cheung J., Perrin D. (2018). Promises and Challenges in the Use of Consumer-Grade Devices for Sleep Monitoring. IEEE Rev. Biomed. Eng..

[128] Kwon S., Kim H., Yeo W.-H. (2021). Recent Advances in Wearable Sensors and Portable Electronics for Sleep Monitoring. iScience.

